# The spatiotemporal control of KatG2 catalase‐peroxidase contributes to the invasiveness of *Fusarium graminearum* in host plants

**DOI:** 10.1111/mpp.12785

**Published:** 2019-03-27

**Authors:** Yan Guo, Shenghua Yao, Tinglu Yuan, Yanzhang Wang, Dong Zhang, Weihua Tang

**Affiliations:** ^1^ National Key Laboratory of Plant Molecular Genetics, Center for Excellence in Molecular Plant Sciences, Institute of Plant Physiology and Ecology Chinese Academy of Sciences Shanghai 200032 China; ^2^ University of the Chinese Academy of Sciences China; ^3^ School of Life Science East China Normal University Shanghai 200062 China; ^4^ Shanghai High School International Division Shanghai 200231 China

**Keywords:** catalase‐peroxidase, *Fusarium graminearum*, hemibiotrophic, reactive oxygen species, temporal and spatial regulation, wheat

## Abstract

Reactive oxygen species (ROS) are involved in the pathogen‐host interactions, and play a Janus‐faced role in the resistance and susceptibility of plants to biotrophic and necrotrophic pathogens. The ascomycete fungus *Fusarium graminearum *causes hazardous wheat Fusarium head blight worldwide. Deletion of the putative secreted catalase‐peroxidase gene in *F. graminearum*, *KatG2*, reduced the virulence in wheat spike infection. However, it remains unclear when and where KatG2 scavenges ROS during the invasion of wheat. In this study, we delineate the change in ROS levels in the transition of the infection phase under microscopic observation. Correspondingly, the pathogen switches its strategy of infection with temporal and spatial regulation of KatG2 to counteract oxidative stress generated by host plant cells. With the native promoter‐driven KatG2‐mRFP strain, we show that KatG2‐mRFP expression was induced *in planta *and accumulated in the infection front region at the early infection stage. In contrast to its ubiquitous cellular localization in runner hyphae, KatG2‐mRFP is exclusively located on the cell wall of invading hyphal cells, especially at the pathogen‐host cellular interface. Using posttranslational modification analysis, we found that asparagine residues at the 238 and 391 positions of KatG2 could be modified by *N*‐glycosylation and that these two residues are required for KatG2 accumulation and cell wall localization *in planta*.

## Introduction

*Fusarium graminearum* is a notorious pathogenic fungus that infects many cereal crops, including wheat and maize. It causes Fusarium head blight on wheat worldwide drastically reducing yield (Dubin, 1997 1996). Its disease cycle starts when airborne ascospores or asexual conidia land on florets during anthesis. The germinated spores produce hyphae growing epiphytically on the surface of floral bracts (named runner hyphae), and then penetrate the surface of lemma and palea. Once inside plant tissues, invasive hyphae extend the colonization from one spikelet to another spikelet through rachis (Brown *et al.*, 2010; Jansen *et al.*,^2005^; Miller *et al.*, 2004; Trail, 2009). In addition to causing significant yield losses, *F. graminearum* also produces mycotoxins such as type B trichothecene deoxynivalenol (DON) in infected spikes and harvested grains; these mycotoxins are required for full virulence on wheat spikes and are a health risk for consumers (Bennett and Klich, 2003; Proctor *et al.*, 1995). *F. graminearum* also causes Gibberella stalk rot in maize, one of the most destructive maize diseases (Chambers, 1987). The *F. graminearum *infection causing various crop diseases is complex and varies (Brown *et al.*, 2010). Although intense research worldwide has made much progress in characterizing the molecular mechanisms underlying *F. graminearum*‐caused diseases, it is still awaiting elucidation of the specific role of manipulating reactive oxygen species (ROS) production and neutralization.

ROS comprising singlet oxygen and reduced forms of oxygen, such as the superoxide anion radical (O_2_^−^) and hydrogen peroxide (H_2_O_2_), are important not only in intracellular redox homeostasis, which regulates cell development, but also in intercellular communication and host‐pathogen interactions (Marschall and Tudzynski, 2016). However, the roles of ROS in plant‐fungal interactions are complicated and dynamic. In addition, the biological roles of ROS often involves being antagonistic to the pathogenicity of biotrophs and necrotrophs. The accumulation of ROS increases host resistance to biotrophic pathogens and susceptibility to necrotrophic pathogens (Barna *et al.*, 2012). Mounting evidence indicates that cellular and subcellular localization and timing of ROS production and/or neutralization, particularly those at the interface between the advancing hyphal front and defending plant cells, can be critical to infection outcome (Segal and Wilson, 2018).

It is well‐documented that plant cells produce ROS (called an ‘oxidative burst’) as one of the earliest cellular responses to pathogen attack. The released ROS can directly damage cell membranes and other components through lipid peroxidation, protein oxidation and DNA mutation, which eventually lead to fungal growth inhibition or even cell death; meanwhile, these ROS can serve as signals to trigger plant immune responses (Torres, 2010). On the fungal side, it is still not clear whether fungal neutralization can significantly reduce oxidative bursts at the infection site. Furthermore, necrotrophic fungal pathogens might also produce extracellular ROS by plasma membrane NADPH oxidases (NOX) to facilitate infection. Therefore, it is plausible that necrotrophic pathogens switch from ROS neutralization to ROS production over changes in infection phases, but the supporting evidence is still limited.

Amongst ROS, H_2_O_2_ is abundant, can diffuse across biological membranes and is heavily involved in fungus‐plant interactions (Gadjev *et al.*, 2008). Catalase catalyzes the decomposition of H_2_O_2_ to water and oxygen and serves as one type of ROS neutralization enzyme (Mishra and Imlay, 2012). Three types of catalases are classified: (i) the typical monofunctional heme‐containing catalases, (ii) bifunctional heme‐containing catalases, (iii) nonheme Mn‐containing catalases (Chelikani *et al.*, 2004). Bifunctional catalases, namely, catalase‐peroxidases (commonly named KatGs), are widely distributed in archaea, bacteria and lower eukaryotes but not in plants and animals. Phylogenetic analyses showed that most fungi have an intracellular KatG protein, KatG1, whereas only phytopathogenic fungi have an extra KatG protein, KatG2, which has an N‐terminal signal sequence for secretion. Therefore, KatG2 is implicated to have a role in host‐pathogen interactions (Zámocký *et al*., 2012). However, the significance of KatG2 contribution to virulence varied amongst fungal pathogens. For example, the KatG2 in the rice blast fungus *Magnaporthe oryzae* exhibits a moderate contribution to infection during the early stages (Tanabe *et al.*, 2011), but in the maize pathogen *Fusarium verticillioides*, virulence was not significantly weakened when *KatG2* gene was deleted (Gao *et al*., 2018). In *F. graminearum*, a genome‐wide functional survey of 31 peroxidase genes identified that the KatG2 encoding gene, *FCA7*, is required for full virulence in wheat spike and increase DON mycotoxin production (Lee *et al.*, 2018), but its catalase activity and subcellular localization have not been verified. Although the gene was first mentioned as *FCA7 *(Lee *et al.*, 2014), to be consistent with the common name based on its biochemical nature as a catalase‐peroxidase, we thereafter call this gene *KatG2.*

In this work, we show that lacking KatG2 led to elevated ROS levels surrounding invasive hyphae during early infection of wheat, with a detailed microscopic analysis of ROS abundance in the paleae and lemmas of wheat infected by *F. graminearum*. Interestingly, the native *KatG2* mRNA level was very low in hyphae cultured on medium but drastically increased during early infection and decreased in later infection stages; on wheat, the KatG2 protein localized in both the cytosol and cell wall in runner hyphae growing outside of wheat tissues, but relocalized to the fungal cell wall preferentially in invasive hyphae growing inside wheat cells. Furthermore, we revealed that KatG2 can be modified by N‐glycosylation at residues N238 and N391, and point mutation analysis showed that N238 and N391 are required for KatG2 accumulation and localization to cell wall in invasive hyphae, and are required for the full virulence of *F. graminearum* on wheat. We propose that *F. graminearum* temporally and spatially controls KatG2 to alleviate plant‐derived ROS in the early invasion phase and facilitate fungal invasion.

## Results

### ROS accumulation levels are spatiotemporally heterogeneous in *F. graminearum*‐infected wheat tissues

**Figure 1:**
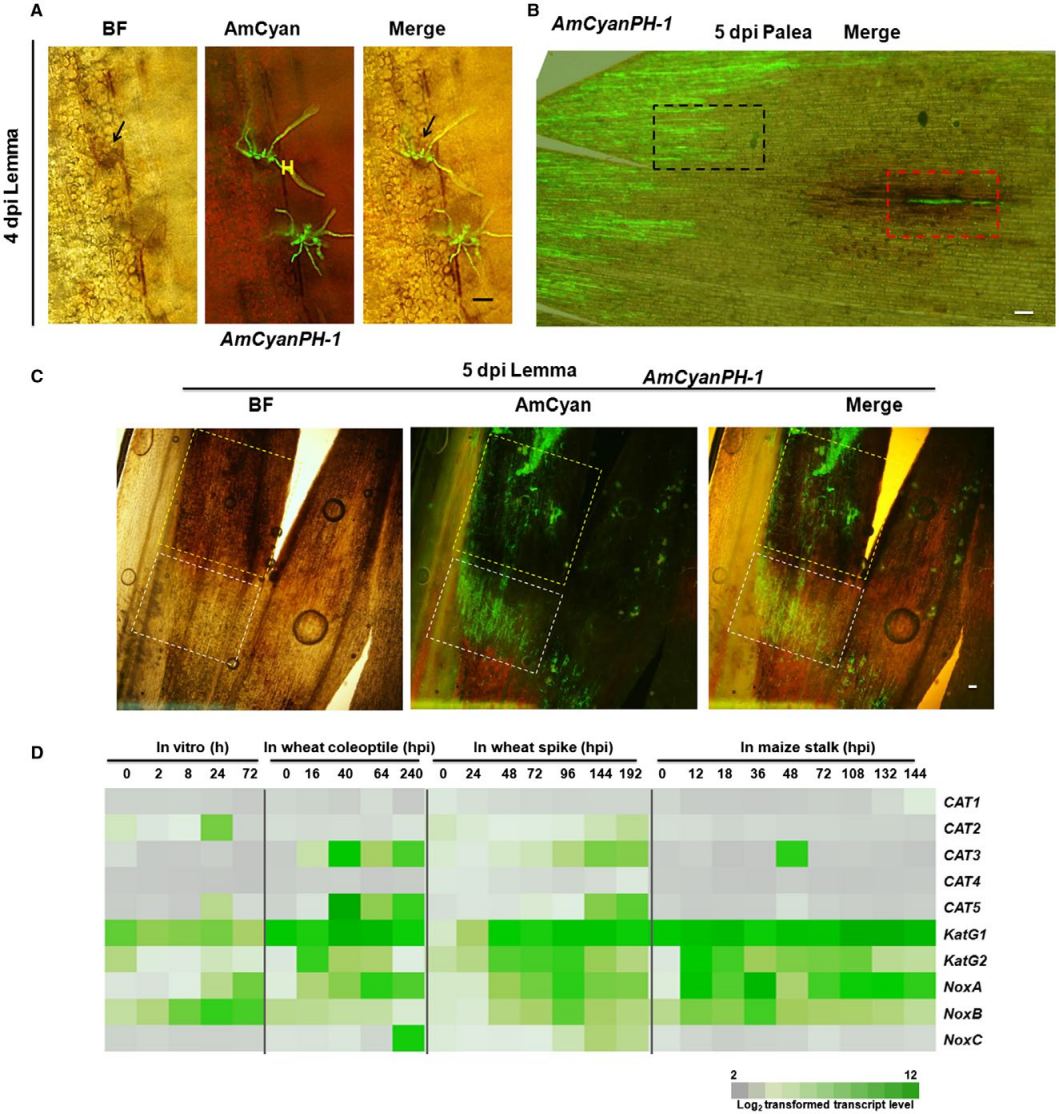
ROS accumulation is different at different positions in *F. graminearum‐*infected wheat tissues. (A)‐(C) DAB staining of AmCyan‐labelled *F. graminearum *infection in the paleae or lemmas of wheat spikes at the indicated time points. Black arrows point to a region with DAB staining signals. The area boxed with black broken lines indicates an exemplified region where many hyphae started from the edge are growing. The area boxed with red broken lines indicates a different region from the same palea where a few isolated hyphae can be observed. The areas boxed with white and yellow broken lines indicate exemplified regions of infection front and rear, respectively. White scale bar = 100 μm. H: *F. graminearum *hyphae. BF: bright field. AmCyan: long‐pass GFP fluorescent channel allowing observation of AmCyan fluorescence (Green) from *F. graminearum *and red fluorescence from chlorophyll (dpi: days post inoculation). (D) Heat map of the gene expression profile of catalase, catalase‐peroxidase and NADPH oxidase genes during *in vitro* growth and infection of wheat coleoptiles, wheat spikes and maize stalks. *NoxA *(FGSG_00739), *NoxB* (FGSG_10807), *NoxC* (FGSG_11195). Transcript levels are log_2_ values (hpi, hours after inoculation).

*F. graminearum *infection of wheat spikes has been highly investigated (Boenisch and Schäfer, 2011; Brown *et al*., 2010; Rittenour and Harris, 2010), but knowledge of how ROS levels change in the infection process is limited. To reveal how ROS level changes are associated with the infection of wheat spike by *F. graminearum*, we inoculated the floral cavity between the palea and lemma of the middle spikelet with conidia of fluorescent protein‐expressing wild‐type *F. graminearum *strain *AmCyanPH‐1*, and then the plants were grown in a high‐humidity growth chamber. At specified time points, the inoculated spikelets were detached, and the infected paleae and lemmas were stained with diaminobenzidine (DAB) to monitor H_2_O_2 _accumulation before microscopic analysis. At 4 days after inoculation (dpi), germinated fungal conidia already produced hyphae, and some fungal hyphae were observed attached to the inner surface of the lemma, mild DAB signal accumulated around the attachment site and were restricted to only a few wheat cells in the vicinity (Fig. 1A). At 5 dpi, the infection was more advanced and the fungal invasion in infected tissues was obviously not synchronized. In general, fungal invasion was faster in the lemmas than in the paleae. On the paleae, many hyphae invaded from the edge and grew in parallel towards inner parts of the paleae (Fig. 1B, the black boxed area as an example). Few DAB signals were visible in these areas around invading hyphae with AmCyan fluorescence. Additionally, on the paleae, some isolated hyphae can be observed in the mid‐region surface, and the wheat cells surrounding these hyphae accumulated overwhelming amount of ROS, as indicated by intensive DAB staining (Fig. 1B, red box). This result showed that at the same time in the same tissue, ROS accumulation levels differed around hyphae with different invasion statuses. At 5 dpi on the lemma, the hyphal networks colonized the majority of this tissue. As shown in Fig. 1C, fungal hyphae occupied most parts of the left lobe of the lemma, with the white boxed area containing hyphae close to advancing front and the yellow boxed area containing hyphae in a relative rear part that are more than 1 mm away from the advancing front. We observed intensive DAB staining towards the rear parts of the infection, while little DAB signal was detected in the infection front (Fig. 1C). The ROS distribution pattern of *F. graminearum *in wheat spikes coincides with the lifestyle transition of a hemibiotrophic pathogen in the host, which has a short biotrophic phase at the infection front, where ROS levels are low; and a necrotrophic phase in the rear parts, where ROS levels are high.

To identify the fungal enzyme responsible for neutralizing ROS at the infection front, we reanalysed *F. graminearum *genome‐encoded catalase and catalase‐peroxidase genes (Table S1) based on phylogenetic analysis, conserved protein domains and subcellular localization prediction (Emanuelsson *et al.*, 2000; Goldberg *et al.*, 2014; Horton *et al.*, 2007; Nielsen *et al.*, 1997). CAT1, CAT2 and CAT3 belong to small‐subunit catalases, CAT4 and CAT5 belong to large‐subunit catalases, KatG1 falls into the intracellular subclade of bifunctional catalases, and KatG2 falls into the extracellular subclade of bifunctional catalases (Fig. S1). We analysed the expression of these genes during *in vitro* culture, infections of wheat coleoptiles and spikes, and maize stalks with published microarray data (Lysoe *et al.*, 2011; Seong *et al.*, 2008; Zhang *et al.*, 2012, 2016). As shown in Fig. 1D, *KatG1* exhibited constitutively high expression levels during plant infection or *in vitro* culture, while *KatG2* expression was significantly up‐regulated at an early stage of plant infection and had decreased expression at later stages of infection. In wheat infection, the *KatG2* expression peak occurred earlier than that of *NoxA*, which encodes an extracellular ROS‐producing enzyme (Fig. 1D). Therefore, we considered KatG2 a candidate responsible for extracellular ROS scavenging during early infection.

### KatG2 contributes to *F. graminearum* pathogenicity by alleviating oxidative stress in the vicinity of invasion hyphae

**Figure 2:**
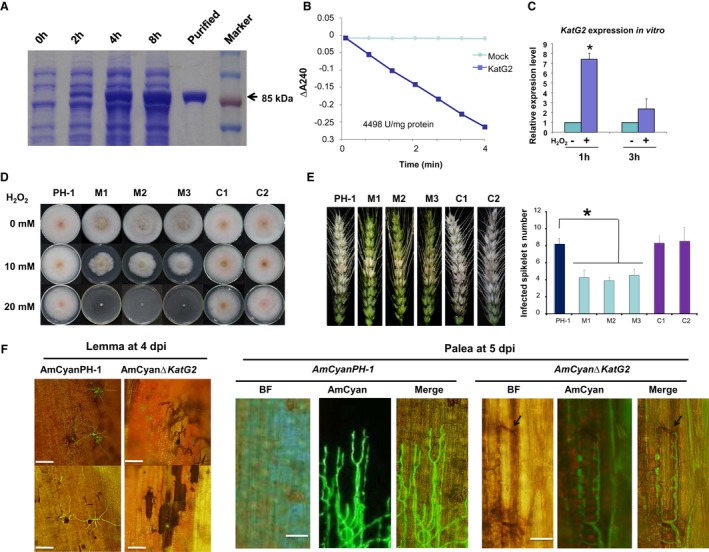
KatG2 alleviates ROS levels surrounding invasive hyphae during host infection. (A) SDS‐PAGE analysis of protein extracts of *E. coli* expressing His‐tag‐fused KatG2 recombinant protein at different times after induction with isopropyl β‐D‐1‐thiogalactopyranoside (IPTG). The size of the KatG2 protein is approximately 85 kDa. (B) The catalase activity of purified recombinant KatG2 was measured. ΔA240 is the decrease in absorbance at 240 nm. (C) KatG2 transcripts were detected by qRT‐PCR at 1 and 3 h after the addition of exogenous H_2_O_2_. Error bars indicate standard deviation. Student’s *t*‐test *P* < 0.05. (D) Δ*KatG2 *mutants showed increased sensitivity to exogenous H_2_O_2_ when cultured *in vitro*. The wild‐type (PH‐1), Δ*KatG2* mutant (M1, M2 and M3) and the complemented strains (C1 and C2) were cultured on CM plates containing different concentrations of H_2_O_2_. (E) Δ*KatG2 *mutants showed reduced virulence in wheat spike infection. Error bars indicate standard errors derived from three independent experiments. Student’s *t*‐test *P* < 0.05. (F) Representative pictures of DAB‐stained wheat spikelets inoculated with conidia of the wild‐type *AmCyanPH‐1* and *AmCyan*Δ*KatG2 *at 4 days post inoculation (dpi) in lemmas and 5 dpi in paleae. Scale bar = 30 μm. Arrows point to DAB signals.

We first examined the catalase activity of KatG2 by expressing recombinant protein *KatG2‐*His in *Escherichia coli* (Fig. 2A). Under our assay conditions, the catalase activity of the purified KatG2 was ~4500 U/mg protein (Fig. 2B), which proved that KatG2 possesses catalase activity *in vitro*. qRT‐PCR analyses showed that *KatG2* expression was increased approximately seven‐fold by 1 h of exposure to H_2_O_2, _and two‐fold after 3 h of exposure to H_2_O_2_ (Fig. 2C), indicating that *KatG2* transcription can be induced by exogenous H_2_O_2_.

Next, we constructed *KatG2* gene deletion mutant strains (M1, M2 and M3) (Fig. S2A‐C) and *KatG2* gene‐complemented lines (C1 and C2) (Fig. S2D). ∆*KatG2* strains grew similarly to the wild‐type strain *in vitro* on complete medium plates and synthetic nutrient‐poor agar plates (Fig. 2D top lane and Fig. S3A). The perithecium formation, ascospore discharge and morphology of ∆*KatG2 *were similar to those of the wild type (Fig. S3B), indicating that mutation of *KatG2 *does not affect vegetative growth and sexual development. However, the colony size of ∆*KatG2* was approximately 80% that of the wild type on plates containing 10 mM H_2_O_2_; the growth of ∆*KatG2* was almost arrested on plates containing 20 mM H_2_O_2_ (Fig. 2D), while complemented strains grew similarly to the wild‐type strain. Moreover, the conidial germination rate of ∆*KatG2* significantly decreased after exposure to 1 mM H_2_O_2_ (Fig. S4). These results showed that ∆*KatG2* mutants were more sensitive to H_2_O_2 _than the wild‐type strain, supporting the role of KatG2 in detoxification of extracellular H_2_O_2_.

We then inoculated wheat spikelets for pathogenicity assessment. On 14 dpi, the wild‐type strain caused symptoms in approximately 8 spikelets per inoculated spike, and ∆*KatG2* caused ~40% fewer diseased spikelets (Fig. 2E), while complemented strains caused a similar number of disease spikelets as the wild‐type strain. This result confirmed that *KatG2* is required for full virulence of *F. graminearum* in wheat head blight infection. In maize stalks, lesions caused by the *KatG2* deletion mutants were ~25% smaller than those caused by the wild‐type strain (Fig. S10). The results showed that KatG2 is also required for full virulence in maize stalk infection.

To assess whether KatG2 affected in overall H_2_O_2_ levels in infected tissues, we compared the accumulation of H_2_O_2_ in wheat spikelets infected by wild‐type and ∆*KatG2* strains using DAB staining (Fig. 2F, S5E and F). At 4 dpi, in the *AmCyan*∆*KatG2*‐infected lemmas, a relatively small quantity of branched hyphae expressing AmCyan was observed, accompanied by strong DAB staining in wheat cells in the vicinity of the hyphae (Fig. 2F), indicating abundant H_2_O_2_ in surrounding host cells. In addition, many conidia exhibited a DAB staining signal but lacked AmCyan fluorescence (Fig. 2F), and further Evans blue staining validated that conidia on paleae lacking fluorescence were inviable (Fig. S5A to D). In contrast, in the wild‐type‐infected lemmas, only a few dead conidia without AmCyan fluorescence were observed, and limited H_2_O_2_ accumulation was observed around branched hyphae of the wild‐type strain (Fig. 2F). In the paleae at 5 dpi, *AmCyanPH‐1* hyphae grew more aggressively than *AmCyan*∆*KatG2* hyphae (Fig. 2F). In the palea inoculated with *AmCyan*∆*KatG2*, H_2_O_2_ accumulation around the front of mutant hyphae was observed (Fig. 2F), while in the palea inoculated with *AmCyanPH‐1*, nearly no accumulation of H_2_O_2 _around the hyphal front was visible (Fig. 2F). These results showed that KatG2 significantly contributed to local ROS levels in the vicinity of hyphae during infection.

### KatG2 was induced specifically *in planta* and expressed in the invasion front of the early invasion stage of *F. graminearum*

**Figure 3:**
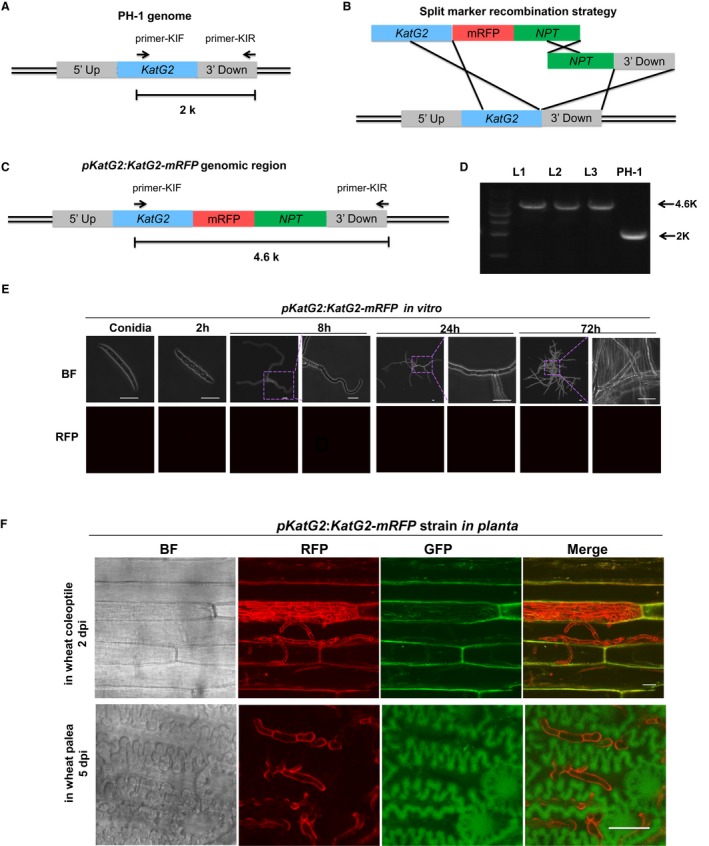
KatG2 expression was induced *in planta*, and KatG2 localized on the fungal cell wall during infection*. *(A) Schematic presentation of the *KatG2* genomic locus in PH‐1. The blue box is the open reading frame of KatG2 in the genome. The grey boxes represent the flanking sequence of KatG2: 5’ UP is upstream of KatG2, and 3’ Down is the 1 kb downstream of KatG2. (B) Split‐marker recombination strategy used to generate *pKatG2:KatG2‐mRFP* knock‐in lines. The red box represents mRFP with the β‐tubulin terminator of *Neurospora crassa. *The green box is the neomycin phosphotransferase (*NPT*) gene, which confers resistance to the antibiotic geneticin. (C) Schematic of *pKatG2::gKatG2‐mRFP *knock‐in lines. (D) Genotype analysis with diagnostic PCR using primers KIF and KIR. The PCR product is 2 kb long in PH‐1 and 4.6 kb long in *pKatG2:KatG2‐mRFP *knock‐in lines. (E) Native promoter‐driven KatG2‐mRFP was not expressed in conidia or hyphae in CM culture. (F) KatG2‐mRFP expressed in the invasion hyphae in coleoptiles and spike paleae. Scale bar = 20 μm.

To gain more insight into the native function of KatG2, we developed a KatG2‐mRFP knock‐in strain of *F. graminearum *by inserting the monomeric red fluorescent protein (mRFP)‐encoding gene just before the stop codon of *KatG2* in the genome, thus allowing visualization of the native expression of *KatG2* *in situ* by mRFP fluorescence signals (Fig. 3A‐C). We named the knock‐in lines *pKatG2:KatG2‐mRFP*. The knock‐in lines were verified with PCR genotyping (Fig. 3D) and sequencing.

With the reporter lines, we found that the fluorescence signals of the mRFP were not visible in conidia and in the germination tube or hyphal branches at 2, 8, 24 and 72 h after cultured in complete medium (Fig. 3E). These results indicated that *KatG2* was not expressed during *in vitro* growth under these conditions. Microscopic analysis of *pKatG2:KatG2‐mRFP*‐inoculated coleoptiles and paleae showed that KatG2‐mRFP signals accumulated at the surface and septa of invading hyphae in coleoptiles at 2 dpi and in paleae at 5 dpi (Fig. 3F), indicating that KatG2 expression was induced *in planta*. The KatG2 expression differences between the *in vitro *and *in planta* conditions were consistent with the microarray data in Fig. 1D.

**Figure 4:**
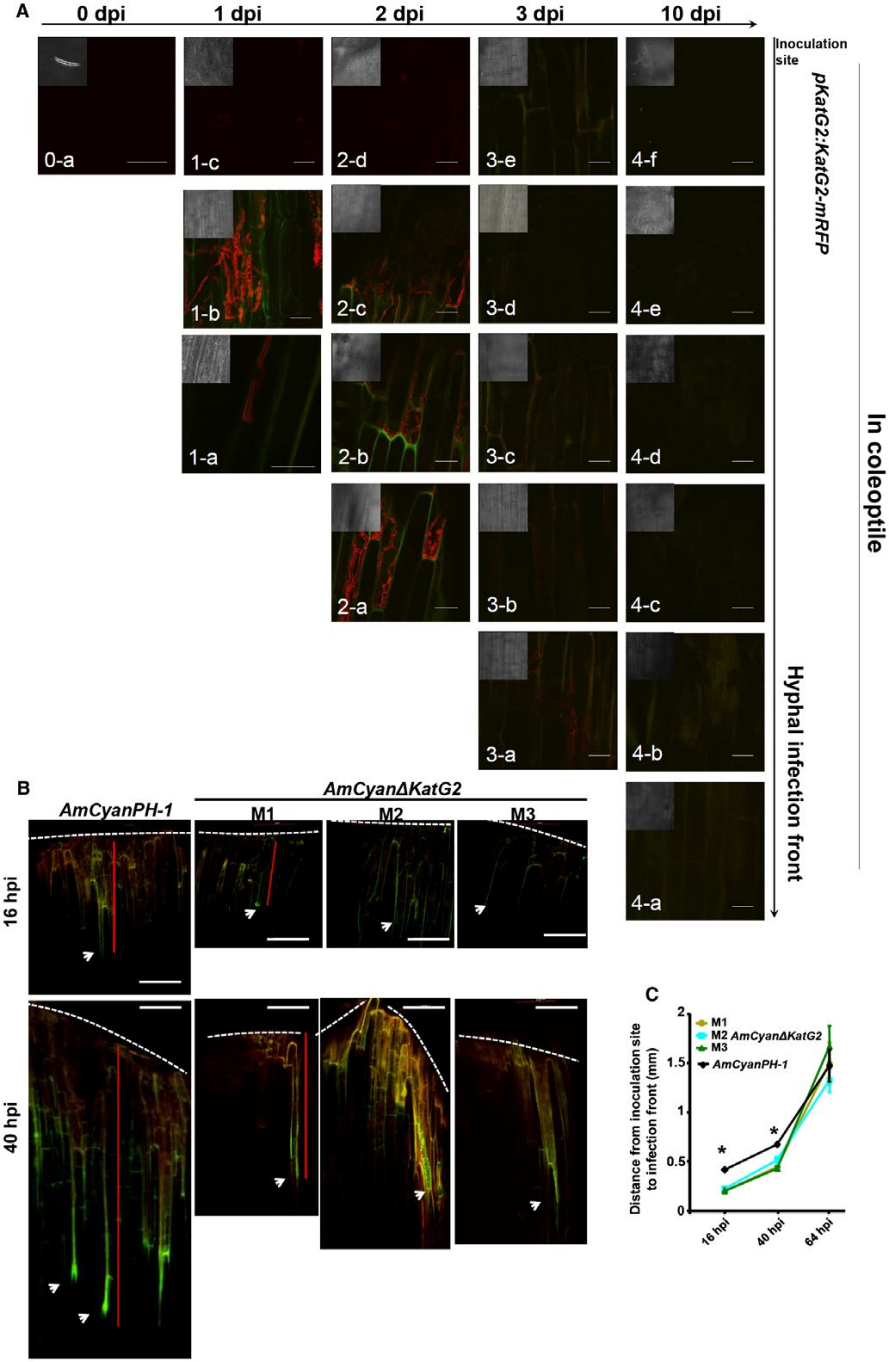
The spatial and temporal expression pattern of KatG2 is consistent with its role during the invasion of coleoptiles. (A) The expression and localization of the native promoter‐driven KatG2‐mRFP over time during infection of the coleoptile. The horizontal axis gives the time after inoculation, and the vertical axis represents the relative position from the inoculation site (top) and invasion front (bottom a) where the pictures were taken. Scale bar = 30 μm. The insets are the corresponding bright field images (in a smaller scale) of the fluorescence microscopic images. (B) Representative pictures of *AmCyan*Δ*KatG2 *mutant and wild‐type hyphae spreading in a coleoptile at 16 hpi and 40 hpi. The arrowhead indicates the hyphal front in the invasion. Broken white lines indicate the edge of the coleoptile, where conidia had been added. Red lines indicate examples of the hyphal spreading distance. Scale bar = 100 μm. (C) Measurements of the coleoptile infection assay at different time points. Error bars indicate standard errors derived from three independent experiments. Student’s *t*‐test *P* < 0.05.

To delineate KatG2 expression during the infection process, we examined the KatG2‐mRFP signal in *pKatG2:KatG2‐mRFP‐*infected coleoptiles at 1 dpi, 2 dpi, 3 dpi and 10 dpi (Fig. 4A). The conidia that just landed on coleoptiles did not show a KatG2‐mRFP signal (Fig. 4A, 0‐a), which is the same as the results shown in Fig. 3E. At 1 dpi and 2 dpi, an intense KatG2‐mRFP signal was observed in germinated conidia and invasion hyphae around the infection front (Fig. 4A, 1‐a,b, 2‐a,b,c), but not in the hyphae network clustered at the inoculation site (Fig. 4A, 1‐c, 2‐d). Notably, we observed that KatG2‐mRFP expression was higher in hyphae close to the advancing front than hyphae close to the rear (Fig. 4A, 2‐c). At the subcellular level, KatG2‐mRFP localized at the septa and fungal cell surface (Fig. 4A, 1‐a,b, 2‐a,b,c). At 3 dpi, KatG2‐mRFP was expressed only in the invasive hyphal front (Fig. 4A, 3‐a) and not in rear parts (Fig. 4A, 3‐b,c,d,e), and the KatG2‐mRFP signal was almost not visible in the hyphae at any position at 10 dpi (Fig. 4A, 3‐a to f). In conclusion, KatG2 expression showed temporal and spatial specificity: temporally, expression was higher in the early infection stages than the late, spatially KatG2 had a higher expression level in the invasive front than in the rear part.

Because KatG2 is mainly expressed during early infection of wheat coleoptile, we examined whether KatG2 deletion led to *F. graminearum *defects in coleoptile invasion during the early stage. We inoculated the coleoptiles with either wild‐type *AmCyanPH‐1* or mutant *AmCyan*∆*KatG2* strains, and measured the distance from invasive hyphae advancing front to the inoculation sites under a fluorescence microscope. At 16 hpi and 40 hpi, the distance that *AmCyan*Δ*KatG2* hyphae advanced in coleoptiles was significantly less than that of the wild type, but the difference diminished at 64 hpi (Fig. 4B and C). This result supports a role for KatG2 in early invasion of wheat coleoptile.

### KatG2 subcellular localization differs in runner hyphae and invasive hyphae

**Figure 5:**
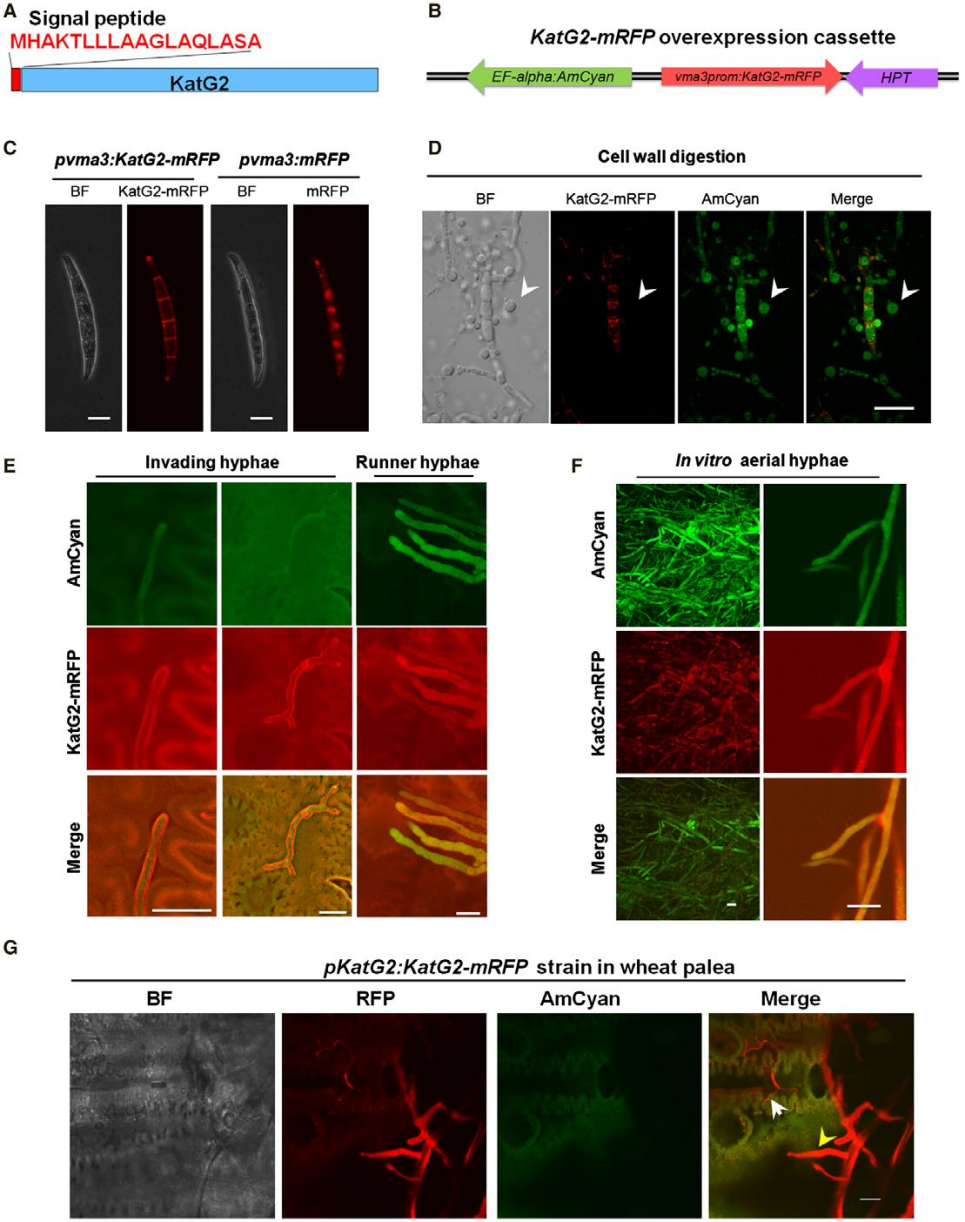
Subcellular localization of KatG2 differs in conidia and hyphae under various growth conditions. (A) Schematic diagram showing the signal peptide at the N‐terminus of KatG2. (B) Schematic map of the plasmid used for constitutive promoter *vma3*‐driven KatG2‐mRFP expression, and the constitutively expressed AmCyan can label the hyphae. (C) KatG2‐mRFP fusion proteins were localized on the cell wall and septa of conidia, while the mRFP signal was strictly localized intracellularly. (D) The 8‐h‐germinated conidia of the strain with *pvma3:KatG2‐mRFP* and *AmCyan* were treated with cell wall lysis solution to produce protoplasts. No KatG2‐mRFP signals were observed in protoplasts lacking cell walls. (E) In wheat spikes, KatG2‐mRFP signals were exclusively located on the cell wall of invading hyphae, while they were distributed ubiquitously in runner hyphae on the surface of wheat. (F) KatG2‐mRFP is localized to the cell surface and intracellularly in runner hyphae *in vitro*. The *pvma3:KatG2‐mRFP *strain was observed after culture on the V8 agar plate for 3 days. (G) KatG2‐mRFP driven with the native promoter showed the same localization pattern in infected wheat paleae at 5 dpi. The yellow arrow indicates runner hyphae, and the white arrow indicates invading hyphae in the intercellular space. Scale bar = 10 μm.

KatG2 has a signal peptide of 18 amino acids in the N‐terminus (Fig. 5A) and was predicted to be secreted (Table S1). To gain more insight into the subcellular localization of KatG2, we constructed a reporter strain named *pvma3:KatG2‐mRFP*;*AmCyan*, which comprises one cassette allowing KatG2‐mRFP expression driven by the strong constitutive *vma3 *promoter (Wechser and Bowman, 1995) and one cassette allowing constitutive expression of AmCyan as an intracellular localization reference (Fig. 5B). During *in vitro *culture, KatG2‐mRFP signals were observed at the periphery and the septa of conidia; by contrast, mRFP alone was observed in the cytoplasm (Fig. 5C). To differentiate whether KatG2‐mRFP localized to the cell wall or plasma membrane of conidia, we treated germinated conidia with cell wall degrading enzymes, and produced protoplasts. KatG2‐mRFP signals were only observed in the septa and peripheral residues of conidia that retained some cell wall and did not completely release protoplasts, and no KatG2‐mRFP signals were observed on the surface of spherical protoplasts lacking cell walls (Fig. 5D). Furthermore, the fluorochrome calcofluor white (CFW) localizes to the fungal cell wall due to its ability to bind chitin (Pringle, 1991). Confocal observation showed that KatG2‐mRFP colocalized with CFW in conidia (Fig. S6).

Next, we observed KatG2 localization in the hyphae of *pvma3:KatG2‐mRFP*;*AmCyan* strain under various conditions. On a 3‐day old culture plate, in the strain, KatG2‐mRFP was ubiquitously localized in the aerial hyphae (Fig. 5F). These results showed that KatG2 localized to the cytoplasm and surface of fungal hyphae during *in vitro* culture. On the infected wheat paleae, interestingly, in the runner hyphae above the surface of palea, KatG2‐mRFP ubiquitously distributed in cytoplasm and cell wall, whereas KatG2‐mRFP mostly gathered on the cell wall in the invading hyphae growing amongst host cells (Fig. 5E). These results showed that KatG2 had different cellular localization in runner hyphae and invading hyphae.

With the *pKatG2:KatG2‐mRFP* strain, we also observed that in one view under microscope, KatG2‐mRFP localized throughout the whole cell in runner hyphae and localized on the cell wall of invading hyphae (Fig. 5G). This evidence confirmed that KatG2‐mRFP signals were mainly located on the cell wall in invading hyphae while they ubiquitously distributed in runner hyphae in the infected spikelets, indicating KatG2 protein subcellular localization is tightly linked to fungal infection status.

### N‐glycosylation modification regulates the localization of KatG2

**Figure 6:**
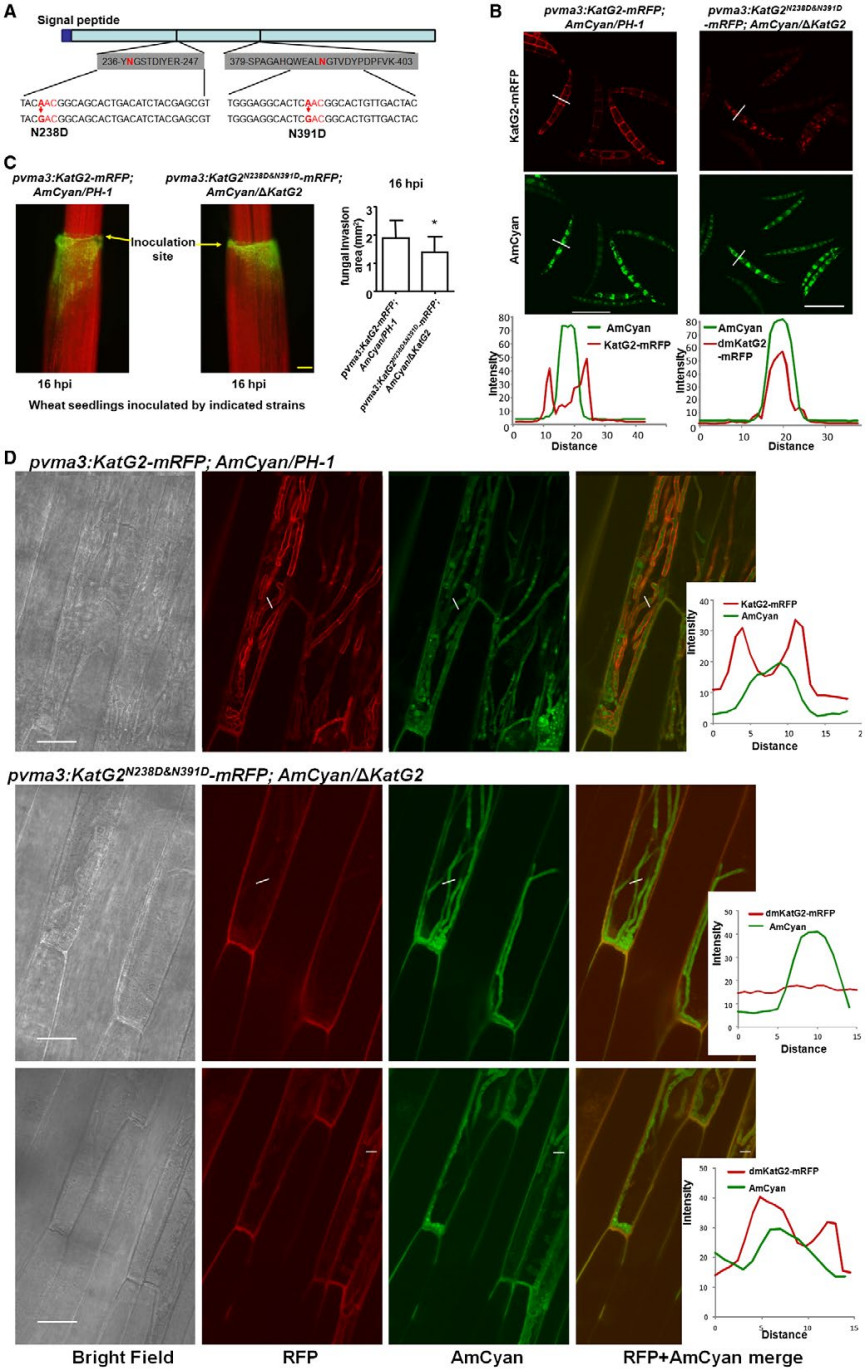
*N*‐glycosylation modification at N238 and N391 is required for cell wall localization of KatG2 in invading hyphae and for full virulence during early infection of wheat coleoptiles. (A) Asparagines at the 238 and 391 positions of KatG2 were identified as *N*‐glycosylated. Each of the sites received PCR‐mediated point mutations with asparagine‐to‐aspartic acid substitutions. (B) KatG2‐mRFP localization in conidia with or without mutation of *N*‐glycosylation sites. Scale bar = 30 μm. Representative fluorescence signal distributions are plotted along the lines shown above. (C) Wheat seedlings infected by the indicated *F. graminearum *strains at 16 h post inoculation. Fungal strains with AmCyan fluorescence were added at the edge of coleoptiles (the middle of images). Images were taken using a fluorescence microscope with a GFP filter, wheat coleoptiles and leaves exhibit red signals due to chlorophyll autofluorescence, and fungal strains exhibiting green signals were observed advancing down from the edge of coleoptile. Yellow scale bar = 400 μm. Fungal invaded areas were measured and charted in the right bar graph. * indicates a significant difference *P* < 0.05 from Student’s* t*‐test. (D) KatG2‐mRFP localization in the invasion hyphae with or without mutation of N‐glycosylation sites. Scale bar = 30 μm. Representative fluorescence signal distributions are plotted along the lines shown left.

The difference in cytoplasm‐cell wall partitioning implied that a layer of KatG2 protein regulation may come from posttranslational modification. Posttranslational modifications of proteins in fungal pathogens have been reported to contribute to their development and virulence (Leach and Brown, 2012). To explore the possibility of protein modification, we constructed a *F. graminearum *strain with a His_6_‐tagged KatG2 (KatG2‐His) protein fusion expression cassette driven by the strong constitutive *vma3 *promoter and then purified KatG2‐His protein from aerial mycelia cultured on a V8 agar plate, infected wheat coleoptiles at 2 dpi and infected wheat spikelets at 14 dpi. Immunoblots with anti‐His antibodies detected fusion proteins with higher molecular weights in infected wheat tissues (coleoptiles and spikes) than in hyphae grown *in vitro*, suggesting a possibility of protein modification during infection (Fig. S7A). KatG2 was predicted to have 3 potential N‐glycosylation motifs (N‐X‐S/T) using NetNGlyc 1.0 Server. To verify and map glycosylation sites, KatG2‐His protein purified from infected wheat tissues digested by trypsin, and the resulting peptides were treated with Peptide‐N‐Glycosidase F (PNGase F) in H_2_^18^O to remove glycan and to incorporate the ^18^O tag into the glycosylated asparagine residues. Using liquid chromatography coupled with tandem mass spectrometry (LC‐MS/MS) matched two ^18^O‐labelled peptides, YNGSTDIYER and SPAGAHQWEALNGTVDYPDPFVK to sequences in KatG2, and within the two peptides, the 238^th^ and 391^st^ asparagine residues of KatG2 (N238 and N239) were identified as N‐glycosylated (Fig. 6A). N238 and N239 are two of the three predicted N‐glycosylation sites and are predicted to be located at the surface of a homodimer of KatG2 (Fig. S8) by homology modelling on the SWISS‐MODEL web server.

To explore whether N‐glycosylation in KatG2 is relevant to its subcellular localization and role in pathogenicity, we constructed single mutations of N238 and N239 and their double mutation in KatG2‐mRFP in the plasmid *vma3pro:KatG2‐mRFP* with asparagine‐to‐aspartic acid (N to D) substitutions (Fig. 6A) and then individually transformed the plasmids into the wild‐type strain. In conidia cultured *in vitro*, the mRFP fusion of the single mutants KatG2^N238D^ and KatG2^N391D^ localized to the cell wall (Fig. S9A), similar to the wild‐type KatG2‐mRFP. However, the mRFP fusion of the double mutant KatG2^N238D&N391D^ accumulated in the cytoplasm and cell wall (Fig. 6B). The fluorescence signal intensity plots showed that the ratio of KatG2^N238D&N391D^‐mRFP on cell wall to that in the cytoplasm was significantly lower than the corresponding ratio comparing of KatG2‐mRFP (Fig. S9B).

To eliminate the influence of the endogenous wild‐type KatG2 in the wild‐type PH‐1 strain, we transformed the plasmid encoding KatG2^N238D&N391D^‐mRFP to Δ*KatG2 *mutant strain and found that more KatG2^N238D&N391D^‐mRFP than KatG2‐mRFP accumulated in the cytoplasm of conidia grown *in vitro* (Fig. 6B). Notably, in the invading hyphae of the Δ*KatG2 *strain KatG2^N238D&N391D^‐mRFP nearly lacked the mRFP signal, in contrast to the KatG2‐mRFP signal, which can be observed mostly on the cell wall in invading hyphae (Fig. 6D), indicating that glycosylation at N238 and N391 is required for accumulation and cell wall localization of KatG2 in invading hyphae. Furthermore, a coleoptile infection assay showed that the Δ*KatG2 *strain expressing KatG2^N238D&N391D^‐mRFP had invaded coleoptile less than the wild‐type strain expressing KatG2‐mRFP by 16 hpi (Fig. 6C). Given that the Δ*KatG2 *strain also invaded coleoptile less than the wild‐type strain at 16 hpi, this result indicated that loss of glycosylation at N238 and N391 abolishes the function of KatG2 in virulence. Therefore, N238 and N391 are required for both the cell wall localization of KatG2 and the full virulence of *F. graminearum*.

**Figure 7:**
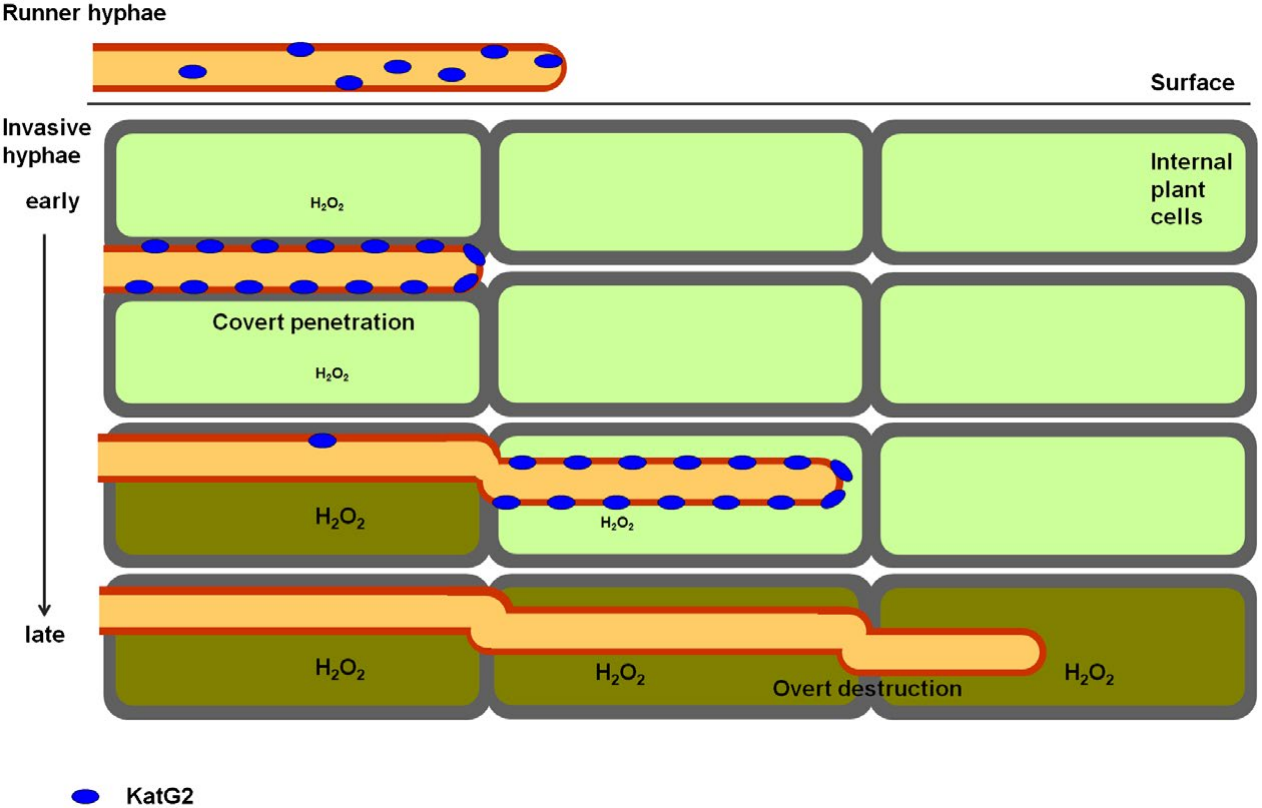
A model for spatial‐temporal regulation of KatG2 contributes to *F. graminearum *hemibiotrophic invasion of host plants. KatG2 protein localized throughout the whole cell in runner hyphae, relocalized to cell wall in invasive hyphae at early biotrophic infection stage, and reduced expression in invasive hyphae at later necrotrophic infection stage.

In conclusion, we propose that spatial‐temporal regulation of KatG2 contributes to *F. graminearum* hemibiotrophic invasion of host plants (Fig. 7). Previous infection stage‐specific gene expression profiles showed that *F. graminearum* increases the expression of extracellular ROS‐scavenging enzyme gene *KatG2* earlier than it increases that of extracellular ROS‐producing enzyme gene *NoxA* during wheat coleoptile infection at the mRNA level (Zhang *et al.*, 2012). In this work, we demonstrate that, at the protein level, expression of ROS‐scavenging KatG2 is higher at the early stage than at the later stages, and that this protein is located throughout the whole cell in runner hyphae outside the plant tissues but relocated in the cell wall of the front parts of the invasive hyphae inside plant tissues, during infection of wheat coleoptile and spikes. The time at which KatG2 is highly expressed and the place at which KatG2 is preferentially cell wall localized correlate with low ROS levels in surrounding wheat cells, and loss of *KatG2* resulted in ROS accumulation around hyphae at the early infection stage, which may cause arrest of hyphal invasion. These results show that KatG2 significantly alleviates host‐derived local ROS levels in advancing fungal fronts to facilitate fungal invasion at the biotrophic phase. In contrast to its ubiquitous subcellular localization in runner hyphae, KatG2‐mRFP was explicitly localized on the cell surface in invading hyphae. N‐glycosylation modification is crucial for KatG2 localization and accumulation during the invasion of wheat.

## Discussion

### KatG2 contributes significantly to *F. graminearum* invasiveness

We showed that the H_2_O_2_‐scavenging protein KatG2 decorates the cell surface of the invading hyphae at the advancing front in wheat (Fig. 3F, 4A, 6D). Its special localization features make the fluorescently tagged KatG2 a useful tool for revealing the cellular behaviour of *F. graminearum* at the pathogen‐host interface during early infection.

With the native promoter‐driven *KatG2‐mRFP* strain, we visualized that KatG2‐mRFP expressed specifically at the hyphal front during the early infection stage (Fig. 4A), in concurrence with the expression pattern of *KatG2 *in the microarray data (Fig. 1D). The attenuated pathogenicity of Δ*KatG2* was restricted before 3 dpi in coleoptiles (Fig. 4B). In the invasion of spikelets, the pathogen has a short biotrophic phase at the infection front and scavenges toxic ROS. The distribution and transition of KatG2 expression support a role for KatG2 in the early biotrophic phase of pathogen infection. It has been argued that *F. graminearum* should be referred to as a hemibiotrophic pathogen or as a necrotrophic pathogen (Ding *et al.*, 2011; Josefsen *et al.*, 2012; Mentges and Bormann, 2015). Microscopy observation combined with DAB staining (Fig. 1A, B and C) supported and advanced the hemibiotrophic proposition. This study improved our understanding in the specific role of manipulating ROS neutralization in virulence, showing that the fungal enzyme really can alleviate host oxidative bursts in the plant–fungal interface. These results are also in line with reported role of ROS accumulation in regulating growth of symbiotic *Epichloë* fungi within its host *Lolium perenne* (Voisey *et al.*, 2016) and in regulating infection of the necrotrophic *Sclerotinia sclerotiorum* in host plants (Williams *et al.*, 2011).

Upstream transcription factors controlling the spatiotemporally expression of *KatG2* are still not clear. The earlier study by Lee *et al. *(2018) found C2H2 zinc finger protein binding motifs and homeobox protein binding motifs in the promoter of *FCA7 *(i.e. *KatG2*), therefore propose a possibility of the direct regulation by GzC2H010 (FGSG_01298, C2H2 zinc finger) and/or GzHOME001 (FGSG_01100, homeobox). However, these two transcription factors were not significantly up‐regulated at 16 hpi during wheat coleoptile infection when *KatG2 *was up‐regulated.

### KatG2 subcellular localization and N‐glycosylation modification regulation

The cell wall of fungi is a network of glycoproteins and polysaccharides (Gow *et al.*, 2017). Many of the glycoproteins are decorated with asparagine (N)‐linked glycosylation. N‐glycosylation is a fundamental posttranslational modification characterized by covalent attachment of an oligosaccharide to asparagine residues containing the N‐X‐S/T sequence, where X cannot be proline (Imperiali *et al.*, 1992). N‐linked glycans perform essential functions in glycoprotein folding, stability, intracellular transport, localization and physiological functions, such as enzyme activity and binding site formation (Chen *et al.*, 2014; Dos Reis Almeida *et al.*, 2011; Everts *et al.*, 1997; Maestre‐Reyna *et al.*, 2015; Wei *et al.*, 2013). N‐Glycoproteome analysis of *F. graminearum* identified 774 N‐glycosylation sites corresponding to 406 proteins and revealed that extracellular protein (47.0%) and membrane protein (16.9%) are highly enriched in N‐glycoprotein (Yu *et al.*, 2016).

N‐glycosylation modifications are involved in protein folding, secretion and protection from degradation. Here, we report that point mutagenesis of *N*‐glycosylation sites in KatG2 leads to its intracellular retention and a decrease in its cell surface accumulation (Fig. 6B). Similar results have been reported in *Paracoccidioides brasiliensis*, where tunicamycin‐mediated inhibition of N‐linked glycosylation of paracoccin led to its intracellular accumulation far from the yeast wall (Dos Reis Almeida *et al.*, 2011). N‐glycosylation of proteins also has been found to function in the virulence of phytopathogen and the regulation of host immunity. For example, in *Magnaporthe oryzae*, the mutant encoding an a‐1,3‐mannosyltransferase ALG3 that is involved in the biosynthesis of N‐glycan of protein N‐glycosylation in the endoplasmic reticulum induces ROS accumulation in plant cells at infection sites, suggesting that N‐glycosylation directly involves host immunity by suppressing ROS production in plant cells during infection. Furthermore, ALG3‐mediated N‐glycosylation of secreted effector Slp1 is required for the protein stability and preventing chitin triggered innate immunity in host plants (Chen *et al.*, 2014). Glycosylation might also contribute to KatG2 conformational stability.

## Experimental Procedures

### Strains and culture conditions

*F. graminearum *wide‐type strain PH‐1 (NRRL 31084) and derived strains in this study were routinely cultured at 25 °C on V8 juice agar and in mung bean broth (MBB) for conidium production as previously described (Zhang *et al.*, 2012).

### Fungal gene expression analyses

The microarray data on conidial germination at different stages (FG7), wheat coleoptile infection (FG19), wheat head blight (FG15) and maize stalk infection were downloaded from PLEXdb and the NCBI Gene Expression Omnibus (Lysoe *et al.*, 2011; Seong *et al.*, 2008; Zhang *et al.*, 2012, 2016). Expression heat maps were generated in R using the ‘heat map’ function of the Bioconductor amp package. The programming code as below: >dat2<‐read.table (file="D:\\KatG2 heatmap microarray data.txt",header=T,sep="\t",row.names=1)>heatcol<‐colorRampPalette(c("grey","honeydew2","darkolivegreen3","green3","green4"))(256)>heatmap(x = as.matrix(dat2), Colv=NA, Rowv=NA, col=heatcol, scale=c("none"), revC=T).

### RNA extraction, reverse transcription and quantitative PCR analysis

Hyphae were cultured for 40 h in YEPD medium and then 0.05% H_2_O_2_ was added. At 1 h and 3 h after addition, hyphae were harvested for RNA extraction. Reverse transcription and quantitative PCR (qPCR) were performed as described previously (Yao *et al.*, 2016). The primers for qPCR are listed in Table S2.

### Construction of deletion mutants and complemented strains

*KatG2 *deletion mutants were generated by the split‐marker recombination procedure (Catlett *et al.*, 2003) Complementation strains were constructed as previous reports (Yao *et al.*, 2016). A genomic fragment containing the promoter and coding region of the *KatG2* gene was amplified and inserted into a vector with a neomycin resistance cassette (Fig. S2D). The primers for generating knockout mutants and complemented strains are listed in Table S2, and the constructed strains are listed in Table S4.

### Infection assay for virulence assessment

Florets of Bobwhite spring wheat were drop‐inoculated, and the disease was rated as described previously (Zhang *et al.*, 2012). For virulence assays in maize stalks, 2‐month‐old maize plants were inoculated as described (He *et al.*, 2016)). Coleoptiles of the wheat zhongyuan 98‐68 were inoculated as described previously (Jia *et al.*, 2017). The infection process was observed with an Olympus BX51 fluorescence microscope or an Olympus Fv 10i confocal microscope. In coleoptile, the distance from the inoculation site to the infection front (Fig. 4C) or the infection area (Fig. 6C and S9D) was quantified with ImageJ software.

### Expression of KatG2 in *E. coli* and catalase activity assay

The full length cDNA of the *KatG2 *gene was cloned into the vector pET‐28a (+). The expression and purification of recombinant proteins were performed as described (Yao *et al.*, 2016). The enzyme activity of KatG2 was tested by monitoring the decomposition of H_2_O_2_ by following a decrease in absorbance at 240 nm measured using an Eon Microplate Spectrophotometer (BioTek, Winooski, VT, USA).

### DAB staining and microscopic observation

The lemmas and paleae of wheat spikelets at 4 and 5 days after inoculation were detached and immersed in 1 mg/ml DAB solution with 0.05% Tween 20. After shaking in 25 °C at 100 r/min for 2 h in the dark, the stained lemmas and paleae were gently rinsed using distilled water and observed under an Olympus BX51 fluorescence microscope. Quantification of DAB staining was evaluated with ImageJ software.

### Analysis of KatG2 subcellular localization

To generate KatG2‐mRFP whose expression was driven by the native KatG2 promoter, a fusion PCR‐based method (Szewczyk *et al.*, 2006) was used with modification to synthesize a construct (Fig. 3B). DNA fragments were transformed into protoplasts of PH‐1. Transformants were confirmed by PCR (Fig. 3C) and sequencing. To test the expression of *pkatG2:KatG2‐mRFP* *in vitro*, a conidial suspension (1 × 10^5^ conidia/mL) was incubated in CM liquid medium shaking at 150 rpm, and the KatG2‐mRFP signal was recorded at the indicated time points. An Olympus Fv 10i confocal microscope was used to collect the fluorescence signal.

To generate constitutively expressed KatG2‐mRFP protein, we cloned the *KatG2* cDNA into the *vma3* promoter‐mRFP vector with the AmCyan unit (Wechser and Bowman, 1995; Zhang *et al.*, 2012). The construct was then transformed into the protoplasts of PH‐1 to obtain transformants.

For partial fungal cell wall removal (Fig. 5D), the germinated conidia with KatG2‐mRFP with AmCyan were treated with 30 ml of cell wall lysis solution containing 750 mg driselase, 12 mg chitinase and 450 mg lysing enzymes.

Coleoptiles of wheat seedlings and spikes of flowering wheat were inoculated with conidial suspension. An Olympus Fv 10i or Fluoview FV 1000 microscope was used for observation. The excitation/emission wavelengths were 584 nm/607 nm for mRFP and 484 nm/507 nm for GFP and AmCyan.

For subcellular localization analysis, a fluorescent localization distribution map was generated with the Olympus Fluoview software (Fig. S6B). The relative KatG2 localization ratio (cell wall/intracellular) was calculated as the ratio of KaG2‐mRFP intensity to AmCyan intensity using AmCyan as a reference (Fig. 6B and S9).

### N‐glycosylation site identification and mutation

KatG2‐HIS fusion protein was purified through Ni‐affinity chromatography (Fig. S7A). The eluted protein was trypsin‐hydrolyzed and then treated with protein‐N‐glycanase (PNGase) in H_2_^18^O to remove the glycan and incorporate the glycosylation‐site‐specific isotope tag. The ^18^O‐labelled peptides were then identified by LC‐MS/MS. The mass spectrometry data were analysed with MaxQuant software (version 1.5.3.17), and the results were searched against the NCBI *F. graminearum* database UniProt_Gibberella_zeae_28973_20180316. Fasta (Fig. S7B). The glycosylation‐modified asparagines of KatG2 were substituted with aspartic acid using point mutation PCR.

### Accession number

KatG2 (NCBI: FGSG_12369)

## Supporting information


**Fig. S1** Neighbour joining tree of catalases (CATs) and catalase peroxidases (KatGs) in *F. graminearum *and other fungi. Af: *Aspergillus fumigatus*; An: *Aspergillus nidulans*; Bm: *Bipolaris maydis*; Sc: *Saccharomyces cerevisiae*; Nc: *Neurospora crassa*; Cn: *Cryptococcus neoformans*; Ca: *Candida albicans*; Mo: *Magnaporthe oryzae*; Fo:* Fusarium oxysporum*; Cg: *Colletotrichum graminicola*. The accession numbers of CATs and KatGs are in Supplemental Table S3. Sequences were aligned with Clustal Omega, and the phylogenetic tree was generated by MEGA6 using Bootstrap confidence values based on 1000 iterations. The evolutionary distances were computed using the P distance method and are in units of the number of amino acid differences per site.

**Fig. S2** Construction of Δ*KatG2* mutants and complemented strains. (A) Restriction maps of genomic fragments containing *KatG2 *in PH 1. (B) Schematic presentation of the split marker recombination strategy used to generate the* KatG2 *gene knockout mutants. (C) and (D) Restriction maps of genomic fragments containing the Δ*KatG2 *allele and the *KatG2* complementation fragment. (E) and (F) Verification of *KatG2 *gene deletion mutants and complemented lines by PCR. A specific product was amplified in the wild type PH 1, and no product was amplified in the Δ*KatG2 *mutants M1, M2 and M3 using *KatG2* gene specific primers in (B); PCR products of different sizes were amplified by primers in the left border (LB) and right border (RB) regions in (A) and (C). (G) and (H) Southern hybridization of PH 1, Δ*KatG2 *mutants and complemented strains. Genomic DNA of each strain was digested with *Pst *I and hybridized with probe A or probe B. Whereas probe A hybridized to the *KatG2 *gene to produce a 7.8 kb band in PH 1 and a 3.2 kb band in complemented strains but did not produce these bands in *ΔKatG2 *mutants, probe B reported the *HPH *gene with a 6.9 kb band in Δ*KatG2 *mutants. LB: 5  flanking sequences of *KatG2*; RB: 3  flanking sequences of *KatG2*; *HPT*: Hygromycin B phosphotransferase gene; *NPT*: Neomycin phosphotransferase gene; M: DNA marker.

**Fig. S3** Δ*KatG2 *mutants showed normal vegetative growth and reproductive development* in vitro*. (A) Wild type (PH 1), Δ*KatG2* mutant (M1, M2, and M3) and complemented strains (C1 and C2) were cultured on synthetic nutrient poor agar (SNA) plates. Photographs were taken 4 days post inoculation (dpi). The colony areas of PH 1, ∆*KatG2 *mutants and complemented strains were examined each day. (B) Perithecium growth on carrot agar plates, ascospore discharge and morphology of ascospores of wild type (PH 1) and Δ*KatG2* mutant (M1) strains. Scale bar = 10 μm.

**Fig. S4** Δ*KatG2 *mutants showed increased sensitivity to exogenous H_2_O_2 _during conidial germination. (A) Conidial germination percentages of PH 1, ∆*KatG2 *mutant (M1) and complemented strains (C1) in liquid CM were examined after 4 and 6 h of exposure to 1 mM H_2_O_2_. Scale bar   50 μm. More than 200 conidia were scored for each line. Asterisks indicate significant differences (Student’s* t‐*test, *P* < 0.05).

**Fig. S5** DAB staining of wheat spikelets inoculated with conidia of the wild type AmCyanPH 1 and AmCyanΔ*KatG2. *(A to D) Evans blue staining was used to check conidial viability. (A) Normal conidia have AmCyan fluorescence and no Evans blue staining, whereas (B) conidia boiled for 10 min lost AmCyan fluorescence and were stained with Evans blue. (C) Conidia of (A) and (B) were mixed and observed in the same field of the microscope. (D) At 4 dpi, in the paleae inoculated with the mutant *AmCyan*∆*KatG2*, many conidia without AmCyan fluorescence were observed, and Evans blue penetrated into the nonviable cells (as arrow indicates). Scale bar   30 μm. (E and F) DAB staining of wheat spikelets inoculated with conidia of wild type *AmCyanPH‐1* and* AmCyan*Δ*KatG2* at 4 dpi in lemmas and 5 dpi in paleae. Error bars indicate standard deviation; the experiment was repeated four times, asterisks indicate significant differences (Student’s *t*‐test, *P* < 0.05), and three asterisks indicate extremely significant differences (Student’s *t*‐test, *P* < 0.001).

**Fig. S6** KatG2 mRFP colocalized with calcofluor white (CFW), a fungal cell wall stain. (A) The fluorescent stain CFW binds cellulose and chitin of conidia. AmCyan fluorescence localized intracellularly, and KatG2 mRFP colocalized on the cell wall and septa with CFW (1 g L). Bar = 10 μm. (B) Fluorescence signal distribution is plotted along the line shown in A.

**Fig. S7** N glycosylation site identification of KatG2. (A) Western blot of purified protein from strain with constitutive promoter driven KatG2 His tagged fusion growing on a V8 agar plate *in vitr*o, in wheat coleoptile and in wheat spike. The black asterisk indicates KatG2 without modification, and the red asterisk indicates the predicted KatG2 modified with glycosylation. (B) Schematic diagrams show the process of detecting the *N* glycosylation with high resolution LC MS MS.

**Fig. S8** N glycosylation sites of KatG2 localized at the surface of the KatG2 tertiary structure. Homology modelling of KatG2 using the SWISS MODEL web server. KatG2 forms a homodimer, and the yellow sites indicate the asparagines at positions 238 and 391, which were predicted to be modified with N glycosylation.

**Fig. S9** Asparagines at positions 238 and 391 of KatG2 regulate the cell wall distribution of KatG2. (A) KatG2 mRFP localization in conidia with or without mutation of N glycosylation sites. To analyse the localization of KatG2 in conidia, the mutation sites were introduced to the plasmid in Fig. 4B, which was then transformed into PH 1 protoplasts. Bar = 30 μm. (B) Statistical analysis of KatG2 localization using AmCyan as a reference. Error bars indicate standard deviation, the experiment was repeated three times, and asterisks indicate significant differences (Student’s *t*‐test, *P* < 0.05).

## References

[mpp12785-bib-0001] Barna, B. , Fodor, J. , Harrach, B.D. , Pogány, M. and Király, Z. (2012) The Janus face of reactive oxygen species in resistance and susceptibility of plants to necrotrophic and biotrophic pathogens. Plant Physiol. Biochem. 59, 37–43. 22321616 10.1016/j.plaphy.2012.01.014

[mpp12785-bib-0002] Bennett, J.W. and Klich, M. (2003) Mycotoxins. Clin. Microbiol. Rev. 16, 497–516. 12857779 10.1128/CMR.16.3.497-516.2003 PMC164220

[mpp12785-bib-0003] Boenisch, M.J. and Schäfer, W. (2011) *Fusarium graminearum* forms mycotoxin producing infection structures on wheat. BMC Plant Biol. 11, 110. 21798058 10.1186/1471-2229-11-110 PMC3166921

[mpp12785-bib-0004] Brown, N.A. , Urban, M. , van de Meene, A.M. and Hammond‐Kosack, K.E. (2010) The infection biology of *Fusarium graminearum*: defining the pathways of spikelet to spikelet colonisation in wheat ears. Fungal Biol. 114, 555–571. 20943167 10.1016/j.funbio.2010.04.006

[mpp12785-bib-0005] Catlett, N. , Lee, B.‐N. , Yoder, O. and Turgeon, B.G. (2003) Split‐marker recombination for efficient targeted deletion of fungal genes. Fungal Genet. Newsl. 50, 9–11.

[mpp12785-bib-0006] Chambers, K.R. (1987) Stalk rot of maize: host‐pathogen interaction. J. Phytopathol. 118, 103–108.

[mpp12785-bib-0007] Chelikani, P. , Fita, I. and Loewen, P.C. (2004) Diversity of structures and properties among catalases. Cell Mol. Life Sci. 61, 192–208. 14745498 10.1007/s00018-003-3206-5 PMC11138816

[mpp12785-bib-0008] Chen, X‐L. , Shi, T. , Yang, J. , Shi, W. , Gao, X. , Chen, D. , Xu, X. , Xu, J‐R. , Talbot, N.J. and Peng, Y‐L. (2014) N‐glycosylation of effector proteins by an alpha‐1,3‐mannosyltransferase is required for the rice blast fungus to evade host innate immunity. Plant Cell, 26, 1360–1376. 24642938 10.1105/tpc.114.123588 PMC4001389

[mpp12785-bib-0009] Ding, L. , Xu, H. , Yi, H. , Yang, L. , Kong, Z. , Zhang, L. , Xue, S. , Jia, H. and Ma, Z. (2011) Resistance to hemi‐biotrophic *F. graminearum* infection is associated with coordinated and ordered expression of diverse defense signaling pathways. PLoS ONE, 6, e19008. 21533105 10.1371/journal.pone.0019008 PMC3080397

[mpp12785-bib-0010] Dos Reis Almeida, F.B. , Carvalho, F.C. , Mariano, V.S. , Alegre, A.C. , Silva Rdo, N. , Hanna, E.S. and Roque‐Barreira, M.C. (2011) Influence of N‐glycosylation on the morphogenesis and growth of *Paracoccidioides brasiliensis* and on the biological activities of yeast proteins. PLoS ONE, 6, e29216. 22216217 10.1371/journal.pone.0029216 PMC3244461

[mpp12785-bib-0011] Dubin, H.J. , Gilchrist, L. , McNab, A. (1997) Fusarium Head Scab: Global Status and Future Prospects: Proceedings of a Workshop Held at CIMMYT, El Batan, Mexico, 13–17 October, 1996. CIMMYT.

[mpp12785-bib-0012] Emanuelsson, O. , Nielsen, H. , Brunak, S. and von Heijne, G. (2000) Predicting subcellular localization of proteins based on their N‐terminal amino acid sequence. J. Mol. Biol. 300, 1005–1016. 10891285 10.1006/jmbi.2000.3903

[mpp12785-bib-0013] Everts, I. , Villmann, C. and Hollmann, M. (1997) N‐glycosylation is not a prerequisite for glutamate receptor function but is essential for lectin modulation. Mol. Pharmacol. 52, 861–873. 9351977 10.1124/mol.52.5.861

[mpp12785-bib-0014] Gadjev, I. , Stone, J.M. and Gechev, T.S. (2008) Programmed cell death in plants: new insights into redox regulation and the role of hydrogen peroxide. Int. Rev. Cell Mol. Biol. 270, 87–144. 19081535 10.1016/S1937-6448(08)01403-2

[mpp12785-bib-0015] Gao, S. , Gold, S.E. and Glenn, A.E. (2018) Characterization of two catalase‐peroxidase‐encoding genes in *Fusarium verticillioides* reveals differential responses to in vitro versus in planta oxidative challenges. Mol. Plant Pathol. 19, 1127–1139. 28802018 10.1111/mpp.12591 PMC6638182

[mpp12785-bib-0016] Goldberg, T. , Hecht, M. , Hamp, T. , Karl, T. , Yachdav, G. , Ahmed, N. , Altermann, U. , Angerer, P. , Ansorge, S. , Balasz, K. , Bernhofer, M. , Betz, A. , Cizmadija, L. , Do, K.T. , Gerke, J. , Greil, R. , Joerdens, V. , Hastreiter, M. , Hembach, K. , Herzog, M. , Kalemanov, M. , Kluge, M. , Meier, A. , Nasir, H. , Neumaier, U. , Prade, V. , Reeb, J. , Sorokoumov, A. , Troshani, I. , Vorberg, S. , Waldraff, S. , Zierer, J. , Nielsen, H. and Rost, B. (2014) LocTree3 prediction of localization. Nucleic Acids Res. 42, W350–355. 24848019 10.1093/nar/gku396 PMC4086075

[mpp12785-bib-0017] Gow, N.A.R. , Latge, J.P. and Munro, C.A. (2017) The fungal cell wall: structure, biosynthesis, and function. Microbiol. Spectr. 5. FUNK‐0035‐2016. doi:10.1128/microbiolspec.FUNK-0035-2016. PMC11687499 28513415

[mpp12785-bib-0018] He, J. , Yuan, T. and Tang, W‐H. (2016) *Fusarium graminearum* maize stalk infection assay and associated microscopic observation protocol. Bio‐Protocol, 6, e2034 10.21769/BioProtoc.2034.

[mpp12785-bib-0019] Horton, P. , Park, K.J. , Obayashi, T. , Fujita, N. , Harada, H. , Adams‐Collier, C. and Nakai, K. (2007) WoLF PSORT: protein localization predictor. Nucleic Acids Res. 35, W585–587. 17517783 10.1093/nar/gkm259 PMC1933216

[mpp12785-bib-0020] Imperiali, B. , Shannon, K. , Unno, M. and Rickert, K. (1992) Mechanistic proposal for asparagine‐linked glycosylation. J. Am. Chem. Soc. 114, 7944–7945.

[mpp12785-bib-0021] Jansen, C. , von Wettstein, D. , Schäfer, W. , Kogel, K.H. , Felk, A. and Maier, F.J. (2005) Infection patterns in barley and wheat spikes inoculated with wild‐type and trichodiene synthase gene disrupted *Fusarium graminearum* . Proc. Natl. Acad. Sci. USA, 102, 16892–16897. 16263921 10.1073/pnas.0508467102 PMC1283850

[mpp12785-bib-0022] Jia, L‐J. , Wang, W‐Q. and Tang, W‐H. (2017) Wheat coleoptile inoculation by *Fusarium graminearum* for large‐scale phenotypic analysis. Bio‐Protocol, 7, e2439 10.21769/BioProtoc.2439. PMC8413627 34541158

[mpp12785-bib-0023] Josefsen, L. , Droce, A. , Sondergaard, T.E. , Sørensen, J.L. , Bormann, J. , Schäfer, W. , Giese, H. and Olsson, S. (2012) Autophagy provides nutrients for nonassimilating fungal structures and is necessary for plant colonization but not for infection in the necrotrophic plant pathogen *Fusarium graminearum* . Autophagy, 8, 326–337. 22240663 10.4161/auto.18705

[mpp12785-bib-0024] Leach, M.D. and Brown, A.J. (2012) Posttranslational modifications of proteins in the pathobiology of medically relevant fungi. Eukaryot Cell, 11, 98–108. 22158711 10.1128/EC.05238-11 PMC3272899

[mpp12785-bib-0025] Lee, Y. , Min, K. , Son, H. , Park, A.R. , Kim, J‐C. , Choi, G.J. and Lee, Y‐W. (2014) ELP3 is involved in sexual and asexual development, virulence, and the oxidative stress response in *Fusarium graminearum* . Mol. Plant‐Microbe Interact. 27, 1344–1355. 25083910 10.1094/MPMI-05-14-0145-R

[mpp12785-bib-0026] Lee, Y. , Son, H. , Shin, J‐Y. , Choi, G‐J. and Lee, Y‐W. (2018) Genome‐wide functional characterization of putative peroxidases in the head blight fungus *Fusarium graminearum* . Mol. Plant Pathol. 19, 715–730. 28387997 10.1111/mpp.12557 PMC6638050

[mpp12785-bib-0027] Lysoe, E. , Seong, K‐Y. and Kistler, H.C. (2011) The transcriptome of *Fusarium graminearum* during the infection of wheat. Mol. Plant‐Microbe Interact. 24, 995–1000. 21585270 10.1094/MPMI-02-11-0038

[mpp12785-bib-0028] Maestre‐Reyna, M. , Liu, W‐C. , Jeng, W‐Y. , Lee, C‐C. , Hsu, C‐A. , Wen, T‐N. , Wang, A‐H. and Shyur, L‐F. (2015) Structural and functional roles of glycosylation in fungal laccase from *Lentinus sp* . PLoS ONE, 10, e0120601. 25849464 10.1371/journal.pone.0120601 PMC4388643

[mpp12785-bib-0029] Marschall, R. and Tudzynski, P. (2016) Reactive oxygen species in development and infection processes. Semin. Cell Dev. Biol. 57, 138–146. 27039026 10.1016/j.semcdb.2016.03.020

[mpp12785-bib-0030] Mentges, M. and Bormann, J. (2015) Real‐time imaging of hydrogen peroxide dynamics in vegetative and pathogenic hyphae of *Fusarium graminearum* . Sci. Rep. 5, 14980. 26446493 10.1038/srep14980 PMC4597226

[mpp12785-bib-0031] Miller, S.S. , Chabot, D.M.P. , Ouellet, T. , Harris, L.J. and Fedak, G. (2004) Use of a *Fusarium graminearum* strain transformed with green fluorescent protein to study infection in wheat (Triticum aestivum). Can. J. Plant Pathol. 26, 453–463.

[mpp12785-bib-0032] Mishra, S. and Imlay, J. (2012) Why do bacteria use so many enzymes to scavenge hydrogen peroxide? Arch. Biochem. Biophys. 525, 145–160. 22609271 10.1016/j.abb.2012.04.014 PMC3413786

[mpp12785-bib-0033] Nielsen, H. , Engelbrecht, J. , Brunak, S. and von Heijne, G. (1997) Identification of prokaryotic and eukaryotic signal peptides and prediction of their cleavage sites. Protein Eng. 10, 1–6. 10.1093/protein/10.1.1 9051728

[mpp12785-bib-0034] Pringle, J.R. (1991) Staining of bud scars and other cell wall chitin with calcofluor. Methods Enzymol. 194, 732–735. 2005820 10.1016/0076-6879(91)94055-h

[mpp12785-bib-0035] Proctor, R.H. , Hohn, T.M. and Mccormick, S.P. (1995) Reduced virulence of *Gibberella zeae* caused by disruption of a trichothecene toxin biosynthetic gene. Mol. Plant‐Microbe Interact. 8, 593–601. 8589414 10.1094/mpmi-8-0593

[mpp12785-bib-0036] Rittenour, W.R. and Harris, S.D. (2010) An in vitro method for the analysis of infection‐related morphogenesis in *Fusarium graminearum* . Mol. Plant Pathol. 11, 361–369. 20447284 10.1111/j.1364-3703.2010.00609.x PMC6640345

[mpp12785-bib-0037] Segal, L.M. and Wilson, R.A. (2018) Reactive oxygen species metabolism and plant‐fungal interactions. Fungal Genet. Biol. 110, 1–9. 29225185 10.1016/j.fgb.2017.12.003

[mpp12785-bib-0038] Seong, K‐Y. , Zhao, X. , Xu, J‐R. , Güldener, U. and Kistler, H.C. (2008) Conidial germination in the filamentous fungus *Fusarium graminearum* . Fungal Genet. Biol. 45, 389–399. 17950638 10.1016/j.fgb.2007.09.002

[mpp12785-bib-0039] Szewczyk, E. , Nayak, T. , Oakley, C.E. , Edgerton, H. , Xiong, Y. , Taheri‐Talesh, N. , Osmani, S.A. and Oakley, B.R. (2006) Fusion PCR and gene targeting in *Aspergillus nidulans* . Nat. Protoc. 1, 3111–3120. 17406574 10.1038/nprot.2006.405

[mpp12785-bib-0040] Tanabe, S. , Ishii‐Minami, N. , Saitoh, K. , Otake, Y. , Kaku, H. , Shibuya, N. , Nishizawa, Y. and Minami, E. (2011) The role of catalase‐peroxidase secreted by *Magnaporthe oryzae* during early infection of rice cells. Mol. Plant‐Microbe Interact. 24, 163–171. 21043575 10.1094/MPMI-07-10-0175

[mpp12785-bib-0041] Torres, M.A. (2010) ROS in biotic interactions. Physiol. Plant, 138, 414–429. 20002601 10.1111/j.1399-3054.2009.01326.x

[mpp12785-bib-0042] Trail, F. (2009) For blighted waves of grain: *Fusarium graminearum* in the postgenomics era. Plant Physiol. 149, 103–110. 19126701 10.1104/pp.108.129684 PMC2613717

[mpp12785-bib-0043] Voisey, C.R. , Christensen, M.T. , Johnson, L.J. , Forester, N.T. , Gagic, M. , Bryan, G.T. , Simpson, W.R. , Fleetwood, D.J. , Card, S.D. , Koolaard, J.P. , Maclean, P.H. and Johnson, R.D. (2016) cAMP signaling regulates synchronised growth of symbiotic *Epichloë* fungi with the host grass *Lolium perenne* . Front. Plant Sci. 7, 1546 10.3389/fpls.2016.01546 27833620 PMC5082231

[mpp12785-bib-0044] Wechser, M.A. and Bowman, B.J. (1995) Regulation of the expression of three housekeeping genes encoding subunits of the Neurospora crassa vacuolar ATPase. Mol. Gen. Genet. 249, 317–327. 7500957 10.1007/BF00290533

[mpp12785-bib-0045] Wei, W. , Chen, L. , Zou, G. , Wang, Q. , Yan, X. , Zhang, J. , Wang, C. and Zhou, Z. (2013) N‐glycosylation affects the proper folding, enzymatic characteristics and production of a fungal β‐glucosidase. Biotechnol. Bioeng. 110, 3075–3084. 24308062 10.1002/bit.24990

[mpp12785-bib-0046] Williams, B. , Kabbage, M. , Kim, H.J. , Britt, R. and Dickman, M.B. (2011) Tipping the balance: *Sclerotinia sclerotiorum* secreted oxalic acid suppresses host defenses by manipulating the host redox environment. PLoS Pathog. 7, e1002107. 21738471 10.1371/journal.ppat.1002107 PMC3128121

[mpp12785-bib-0047] Yao, S‐H. , Guo, Y. , Wang, Y‐Z. , Zhang, D. , Xu, L. and Tang, W‐H. (2016) A cytoplasmic Cu‐Zn superoxide dismutase SOD1 contributes to hyphal growth and virulence of *Fusarium graminearum* . Fungal Genet. Biol. 91, 32–42. 27037138 10.1016/j.fgb.2016.03.006

[mpp12785-bib-0048] Yu, L. , He, H. , Hu, Z. and Ma, Z. (2016) Comprehensive quantification of N‐glycoproteome in *Fusarium graminearum* reveals intensive glycosylation changes against fungicide. J. Proteomics, 142, 82–90. 27180282 10.1016/j.jprot.2016.05.011

[mpp12785-bib-0049] Zámocký, M. , Gasselhuber, B. , Furtmüller, P.G. and Obinger, C. (2012) Molecular evolution of hydrogen peroxide degrading enzymes. Arch. Biochem. Biophys. 525, 131–144. 22330759 10.1016/j.abb.2012.01.017 PMC3523812

[mpp12785-bib-0050] Zhang, X‐W. , Jia, L‐J. , Zhang, Y. , Jiang, G. , Li, X. , Zhang, D. and Tang, W‐H. (2012) In planta stage‐specific fungal gene profiling elucidates the molecular strategies of *Fusarium graminearum* growing inside wheat coleoptiles. Plant Cell, 24, 5159–5176. 23266949 10.1105/tpc.112.105957 PMC3556981

[mpp12785-bib-0051] Zhang, Y. , He, J. , Jia, L‐J. , Yuan, T‐L. , Zhang, D. , Guo, Y. , Wang, Y. and Tang, W‐H. (2016) Cellular tracking and gene profiling of *Fusarium graminearum* during maize stalk rot Disease development elucidates its strategies in confronting phosphorus limitation in the host apoplast. PLoS Pathog. 12, e1005485. 26974960 10.1371/journal.ppat.1005485 PMC4790934

