# Supplementary material for: The spatiotemporal control of KatG2 catalase‐peroxidase contributes to the invasiveness of Fusarium graminearum in host plants
Source: Mol Plant Pathol. 2019 Mar 27;20(5):685–700. doi: 10.1111/mpp.12785 (PMC6637876; doi:10.1111/mpp.12785)
Supplement: Supplementary file 1 — Fig. S1 Neighbour joining tree of catalases (CATs) and catalase peroxidases (KatGs) in F. graminearum and other fungi. Af: Aspergillus fumigatus; An: Aspergillus nidulans; Bm: Bipolaris maydis; Sc: Saccharomyces cerevisiae; Nc: Neurospora crassa; Cn: Cryptococcus neoformans; Ca: Candida albicans; Mo: Magnaporthe oryzae; Fo: Fusarium oxysporum; Cg: Colletotrichum graminicola. The accession numbers of CATs and KatGs are in Supplemental Table S3. Sequences were aligned with Clustal Omega, and the phylogenetic tree was generated by MEGA6 using Bootstrap confidence values based on 1000 iterations. The evolutionary distances were computed using the P distance method and are in units of the number of amino acid differences per site. [file MPP-20-685-s001.pdf]

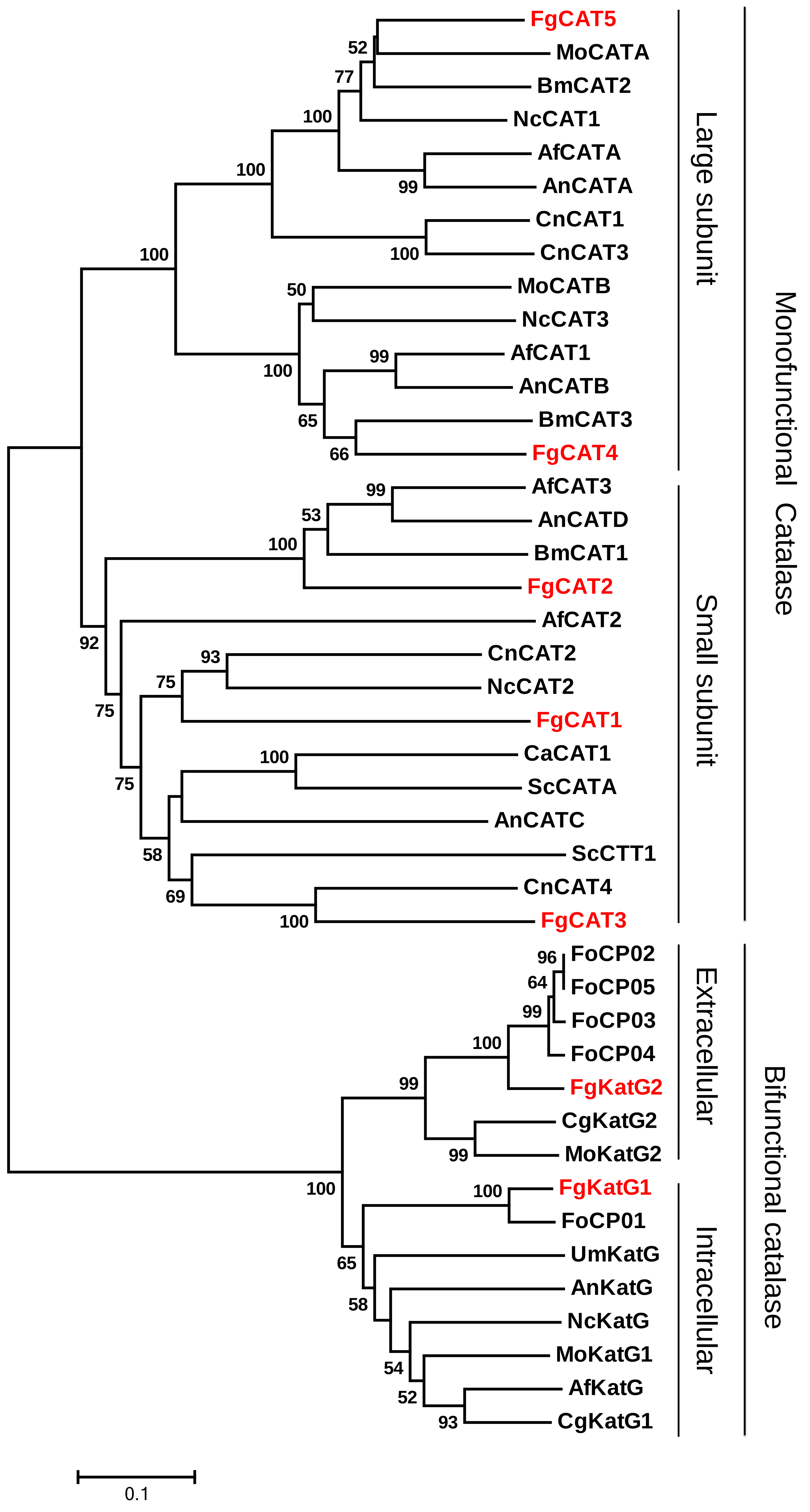

Fig. S1 Neighbor-joining tree of catalases (CATs) and catalase-peroxidases (KatGs) in *F. graminearum* and other fungi. Af: *Aspergillus fumigatus*; An: *Aspergillus nidulans*; Bm: *Bipolaris maydis*; Sc: *Saccharomyces cerevisiae*; Nc: *Neurospora crassa*; Cn: *Cryptococcus neoformans*; Ca: *Candida albicans*; Mo: *Magnaporthe oryzae*; Fo: *Fusarium oxysporum*; Cg: *Colletotrichum graminicola*. The accession numbers of CATs and KatGs are in Supplemental Table S3. Sequences were aligned with Clustal Omega, and the phylogenetic tree was generated by MEGA6 using Bootstrap confidence values based on 1000 iterations. The evolutionary distances were computed using the P-distance method and are in units of the number of amino acid differences per site.
