# Supplementary material for: The spatiotemporal control of KatG2 catalase‐peroxidase contributes to the invasiveness of Fusarium graminearum in host plants
Source: Mol Plant Pathol. 2019 Mar 27;20(5):685–700. doi: 10.1111/mpp.12785 (PMC6637876; doi:10.1111/mpp.12785)
Supplement: Supplementary file 2 — Fig. S2 Construction of ΔKatG2 mutants and complemented strains. (A) Restriction maps of genomic fragments containing KatG2 in PH 1. (B) Schematic presentation of the split marker recombination strategy used to generate the KatG2 gene knockout mutants. (C) and (D) Restriction maps of genomic fragments containing the ΔKatG2 allele and the KatG2 complementation fragment. (E) and (F) Verification of KatG2 gene deletion mutants and complemented lines by PCR. A specific product was amplified in the wild type PH 1, and no product was amplified in the ΔKatG2 mutants M1, M2 and M3 using KatG2 gene specific primers in (B); PCR products of different sizes were amplified by primers in the left border (LB) and right border (RB) regions in (A) and (C). (G) and (H) Southern hybridization of PH 1, ΔKatG2 mutants and complemented strains. Genomic DNA of each strain was digested with Pst I and hybridized with probe A or probe B. Whereas probe A hybridized to the KatG2 gene to produce a 7.8 kb band in PH 1 and a 3.2 kb band in complemented strains but did not produce these bands in ΔKatG2 mutants, probe B reported the HPH gene with a 6.9 kb band in ΔKatG2 mutants. LB: 5 flanking sequences of KatG2; RB: 3 flanking sequences of KatG2; HPT: Hygromycin B phosphotransferase gene; NPT: Neomycin phosphotransferase gene; M: DNA marker. [file MPP-20-685-s002.pdf]

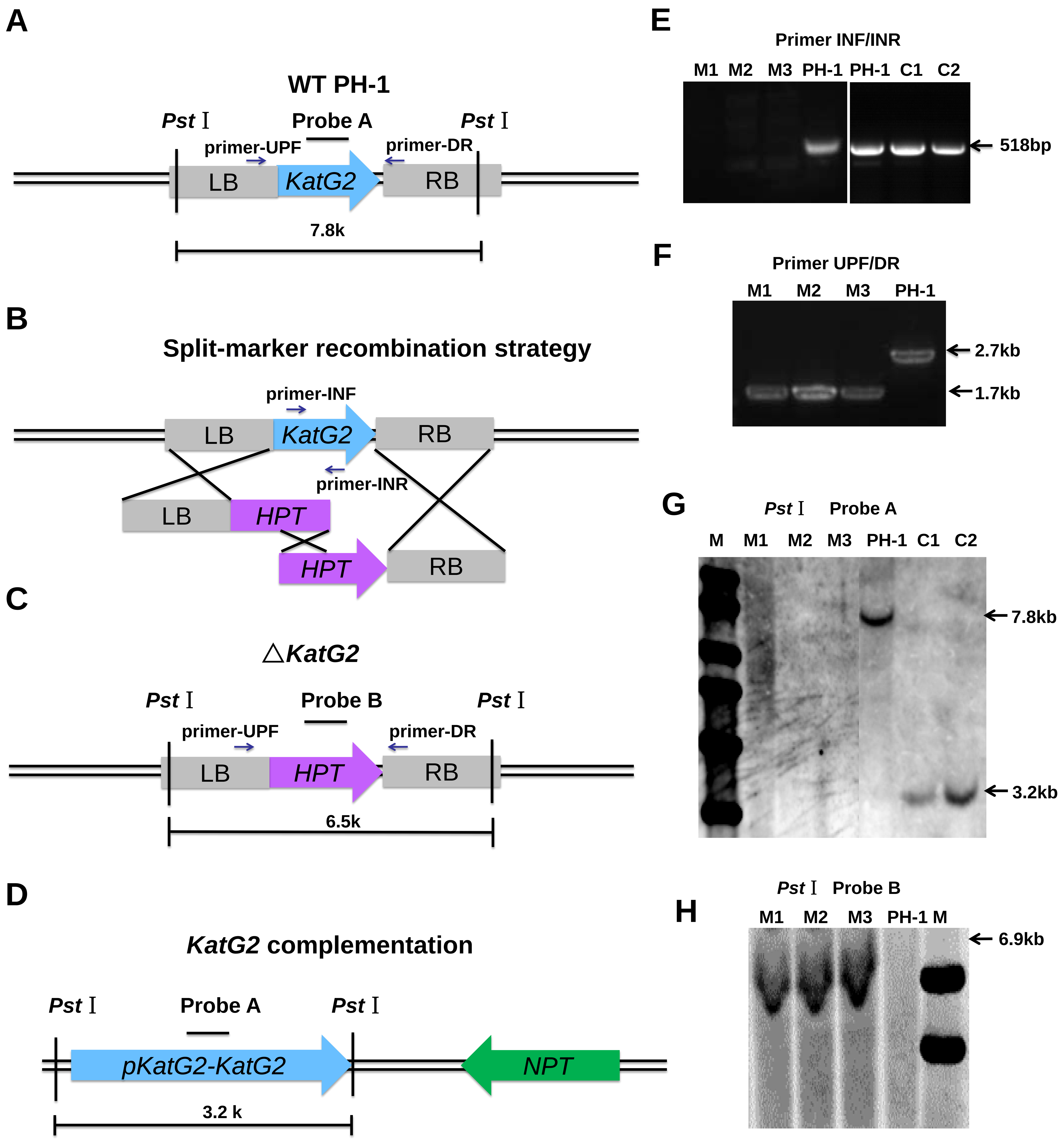

Fig. S2 Construction of Δ*KatG2* mutants and complemented strains. (A) Restriction maps of genomic fragments containing *KatG2* in PH-1. (B) Schematic presentation of the split-marker recombination strategy used to generate the *KatG2* gene knockout mutants. (C) and (D) Restriction maps of genomic fragments containing the Δ*KatG2* allele and the *KatG2* complementation fragment. (E) and (F) Verification of *KatG2* gene deletion mutants and complemented lines by PCR. A specific product was amplified in the wild-type PH-1, and no product was amplified in the Δ *KatG2* mutants M1, M2 and M3 using *KatG2* gene-specific primers in (B); PCR products of different sizes were amplified by primers in the left border (LB) and right border (RB) regions in (A) and (C). (G) and (H) Southern hybridization of PH-1, Δ *KatG2* mutants and complemented strains. Genomic DNA of each strain was digested with *Pst* I and hybridized with probe A or probe B. Whereas probe A hybridized to the *KatG2* gene to produce a 7.8-kb band in PH-1 and a 3.2-kb band in complemented strains but did not produce these bands in Δ *KatG2* mutants, probe B reported the *HPH* gene with a 6.9-kb band in Δ *KatG2* mutants. LB: 5' flanking sequences of *KatG2*; RB: 3' flanking sequences of *KatG2*; *HPT*: Hygromycin B phosphotransferase gene; *NPT*: Neomycin phosphotransferase gene; M: DNA marker.
