# Supplementary material for: The spatiotemporal control of KatG2 catalase‐peroxidase contributes to the invasiveness of Fusarium graminearum in host plants
Source: Mol Plant Pathol. 2019 Mar 27;20(5):685–700. doi: 10.1111/mpp.12785 (PMC6637876; doi:10.1111/mpp.12785)
Supplement: Supplementary file 3 — Fig. S3 ΔKatG2 mutants showed normal vegetative growth and reproductive development in vitro. (A) Wild type (PH 1), ΔKatG2 mutant (M1, M2, and M3) and complemented strains (C1 and C2) were cultured on synthetic nutrient poor agar (SNA) plates. Photographs were taken 4 days post inoculation (dpi). The colony areas of PH 1, ∆KatG2 mutants and complemented strains were examined each day. (B) Perithecium growth on carrot agar plates, ascospore discharge and morphology of ascospores of wild type (PH 1) and ΔKatG2 mutant (M1) strains. Scale bar = 10 μm. [file MPP-20-685-s003.pdf]

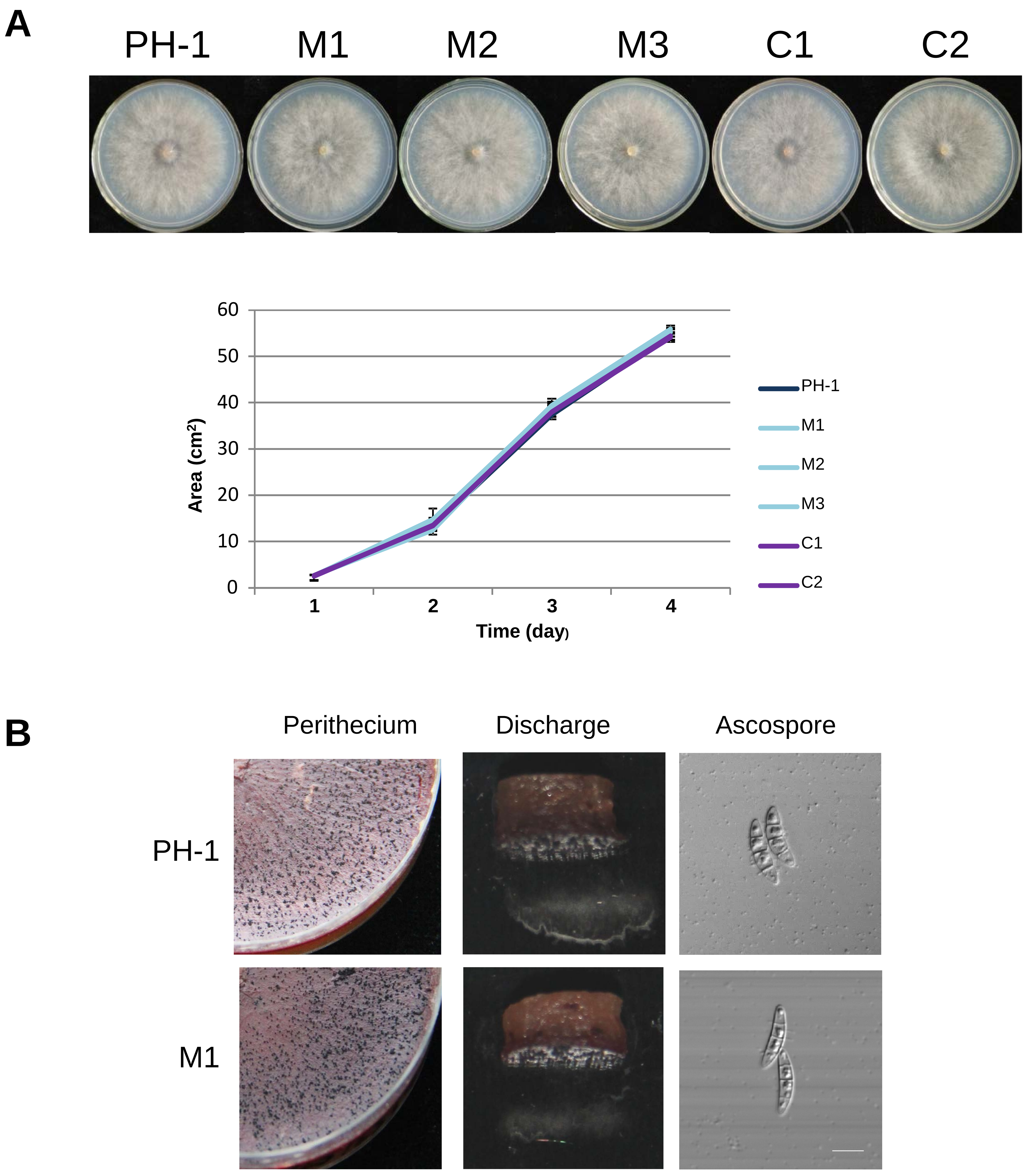

Fig. S3 ***ΔKatG2*** **mutants showed normal vegetative growth and reproductive development** ***in vitro***. (A) (A) Wild-type (PH-1), Δ*KatG2* mutant (M1, M2, and M3) and complemented strains (C1 and C2) were cultured on synthetic nutrient-poor agar (SNA) plates. Photographs were taken four days post inoculation (dpi). The colony areas of PH-1, Δ*KatG2* mutants and complemented strains were examined each day. (B) Perithecium growth on carrot agar plates, ascospore discharge and morphology of ascospores of wild-type (PH-1) and Δ*KatG2* mutant (M1) strains. Scale bar=10 μm.
