# Supplementary material for: The spatiotemporal control of KatG2 catalase‐peroxidase contributes to the invasiveness of Fusarium graminearum in host plants
Source: Mol Plant Pathol. 2019 Mar 27;20(5):685–700. doi: 10.1111/mpp.12785 (PMC6637876; doi:10.1111/mpp.12785)
Supplement: Supplementary file 4 — Fig. S4 ΔKatG2 mutants showed increased sensitivity to exogenous H2O2 during conidial germination. (A) Conidial germination percentages of PH 1, ∆KatG2 mutant (M1) and complemented strains (C1) in liquid CM were examined after 4 and 6 h of exposure to 1 mM H2O2. Scale bar 50 μm. More than 200 conidia were scored for each line. Asterisks indicate significant differences (Student’s t‐test, P < 0.05). [file MPP-20-685-s004.pdf]

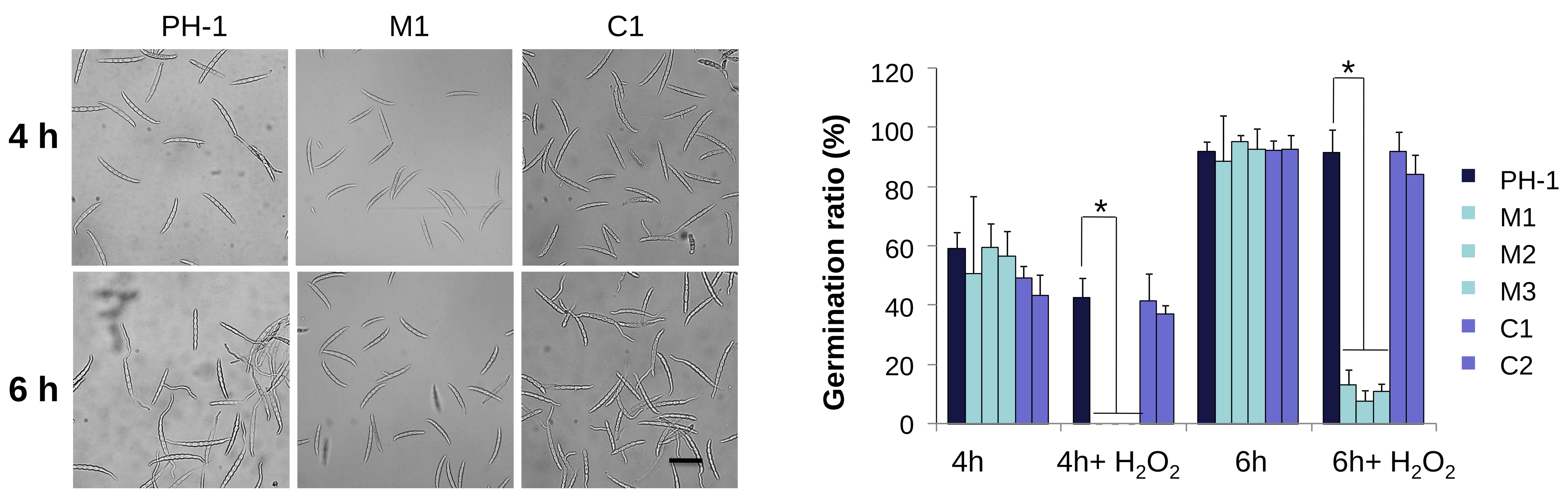

Fig. S4 Δ*KatG2* mutants showed increased sensitivity to exogenous H_2O_2 during conidial germination. (A) Conidial germination percentages of PH-1, Δ*KatG2* mutant (M1) and complemented strains (C1) in liquid CM were examined after 4 and 6 h of exposure to 1 mM H_2O_2. Scale bar=50 μm. More than 200 conidia were scored for each line. Asterisks indicate significant differences (Student's *t*-test, $P<0.05$).
