# Supplementary material for: The spatiotemporal control of KatG2 catalase‐peroxidase contributes to the invasiveness of Fusarium graminearum in host plants
Source: Mol Plant Pathol. 2019 Mar 27;20(5):685–700. doi: 10.1111/mpp.12785 (PMC6637876; doi:10.1111/mpp.12785)
Supplement: Supplementary file 5 — Fig. S5 DAB staining of wheat spikelets inoculated with conidia of the wild type AmCyanPH 1 and AmCyanΔKatG2. (A to D) Evans blue staining was used to check conidial viability. (A) Normal conidia have AmCyan fluorescence and no Evans blue staining, whereas (B) conidia boiled for 10 min lost AmCyan fluorescence and were stained with Evans blue. (C) Conidia of (A) and (B) were mixed and observed in the same field of the microscope. (D) At 4 dpi, in the paleae inoculated with the mutant AmCyan∆KatG2, many conidia without AmCyan fluorescence were observed, and Evans blue penetrated into the nonviable cells (as arrow indicates). Scale bar 30 μm. (E and F) DAB staining of wheat spikelets inoculated with conidia of wild type AmCyanPH‐1 and AmCyanΔKatG2 at 4 dpi in lemmas and 5 dpi in paleae. Error bars indicate standard deviation; the experiment was repeated four times, asterisks indicate significant differences (Student’s t‐test, P < 0.05), and three asterisks indicate extremely significant differences (Student’s t‐test, P < 0.001). [file MPP-20-685-s005.pdf]

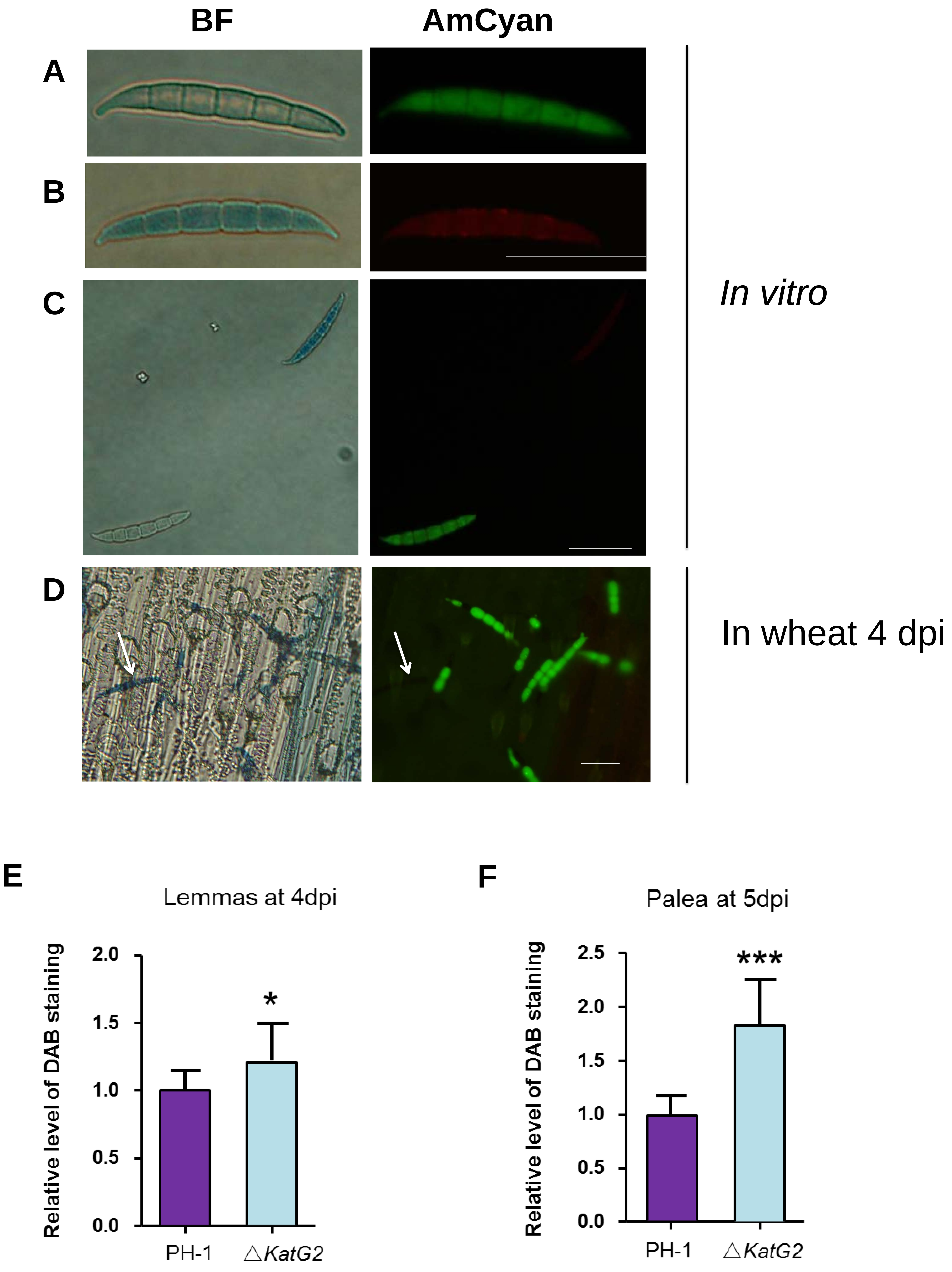

Fig. S5 DAB staining of wheat spikelets inoculated with conidia of the wild-type *AmCyanPH-1* and *AmCyanΔKatG2*. (A to D) Evans blue staining was used to check conidial viability. (A) Normal conidia have AmCyan fluorescence and no Evans blue staining, whereas (B) conidia boiled for 10 minutes lost AmCyan fluorescence and were stained with Evans blue. (C) Conidia of (A) and (B) were mixed and observed in the same field of the microscope. (D) At 4 dpi, in the paleae inoculated with the mutant *AmCyanΔKatG2*, many conidia without AmCyan fluorescence were observed, and Evans blue penetrated into the nonviable cells (as arrow indicates). Scale bar=30 µm. (E and F) DAB staining of wheat spikelets inoculated with conidia of wild-type *AmCyanPH-1* and *AmCyanΔKatG2* at 4 dpi in lemmas and 5 dpi in paleae. Error bars indicate standard deviation; the experiment was repeated four times, asterisks indicate significant differences (Student's *t*-test P<0.05), and three asterisks indicate extremely significant differences (Student's *t*-test P<0.001).
