# Supplementary material for: The spatiotemporal control of KatG2 catalase‐peroxidase contributes to the invasiveness of Fusarium graminearum in host plants
Source: Mol Plant Pathol. 2019 Mar 27;20(5):685–700. doi: 10.1111/mpp.12785 (PMC6637876; doi:10.1111/mpp.12785)
Supplement: Supplementary file 6 — Fig. S6 KatG2 mRFP colocalized with calcofluor white (CFW), a fungal cell wall stain. (A) The fluorescent stain CFW binds cellulose and chitin of conidia. AmCyan fluorescence localized intracellularly, and KatG2 mRFP colocalized on the cell wall and septa with CFW (1 g L). Bar = 10 μm. (B) Fluorescence signal distribution is plotted along the line shown in A. [file MPP-20-685-s006.pdf]

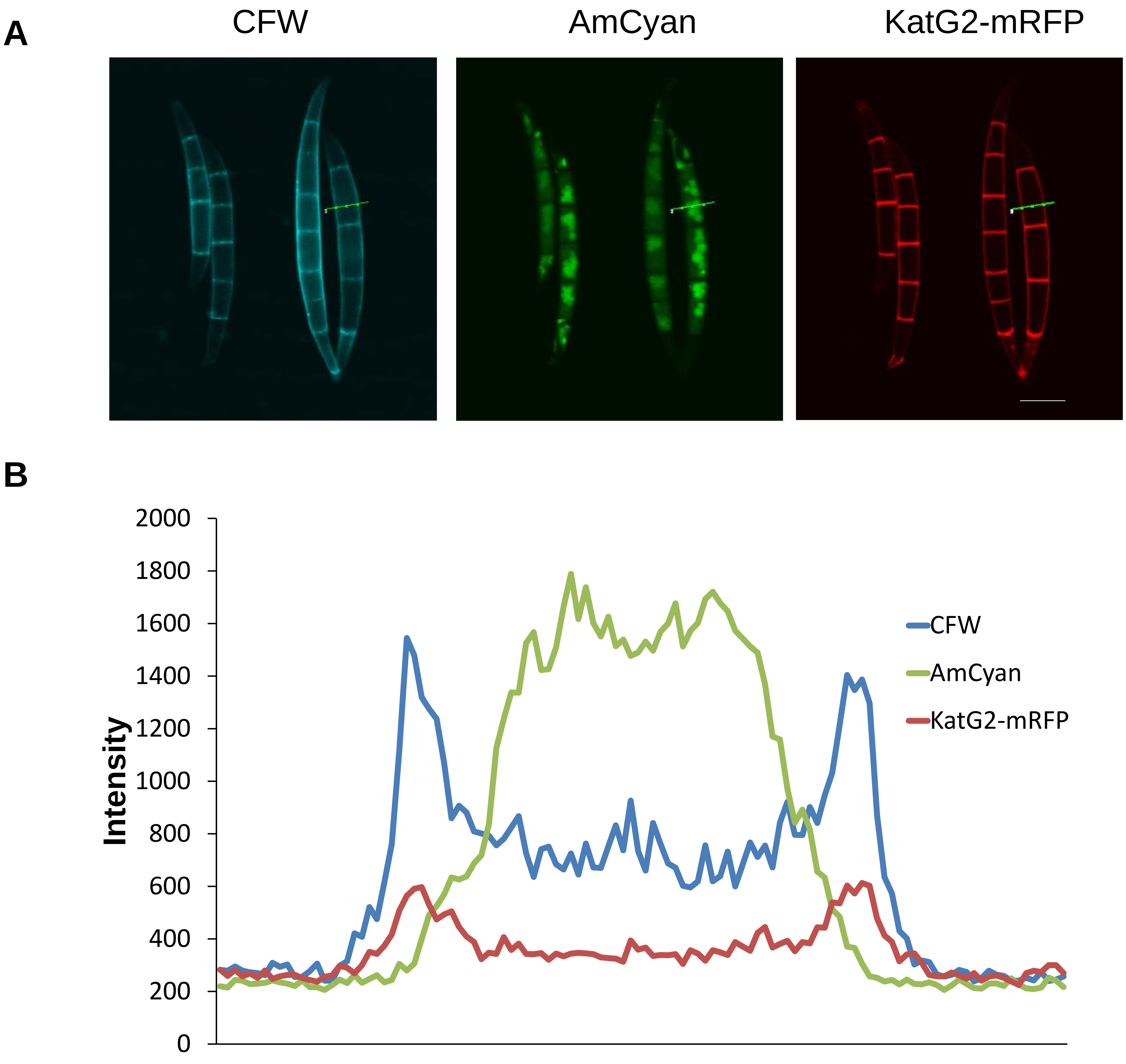

Fig. S6 KatG2-mRFP colocalized with calcofluor white, a fungal cell wall stain. (A) The fluorescent stain calcofluor white (CFW) binds cellulose and chitin of conidia. AmCyan fluorescence localized intracellularly, and KatG2-mRFP colocalized on the cell wall and septa with CFW (1 g/L). Bar=10 μm. (B) Fluorescence signal distribution is plotted along the line shown in A.
