# Supplementary material for: The spatiotemporal control of KatG2 catalase‐peroxidase contributes to the invasiveness of Fusarium graminearum in host plants
Source: Mol Plant Pathol. 2019 Mar 27;20(5):685–700. doi: 10.1111/mpp.12785 (PMC6637876; doi:10.1111/mpp.12785)
Supplement: Supplementary file 7 — Fig. S7 N glycosylation site identification of KatG2. (A) Western blot of purified protein from strain with constitutive promoter driven KatG2 His tagged fusion growing on a V8 agar plate in vitro, in wheat coleoptile and in wheat spike. The black asterisk indicates KatG2 without modification, and the red asterisk indicates the predicted KatG2 modified with glycosylation. (B) Schematic diagrams show the process of detecting the N glycosylation with high resolution LC MS MS. [file MPP-20-685-s007.pdf]

A

B

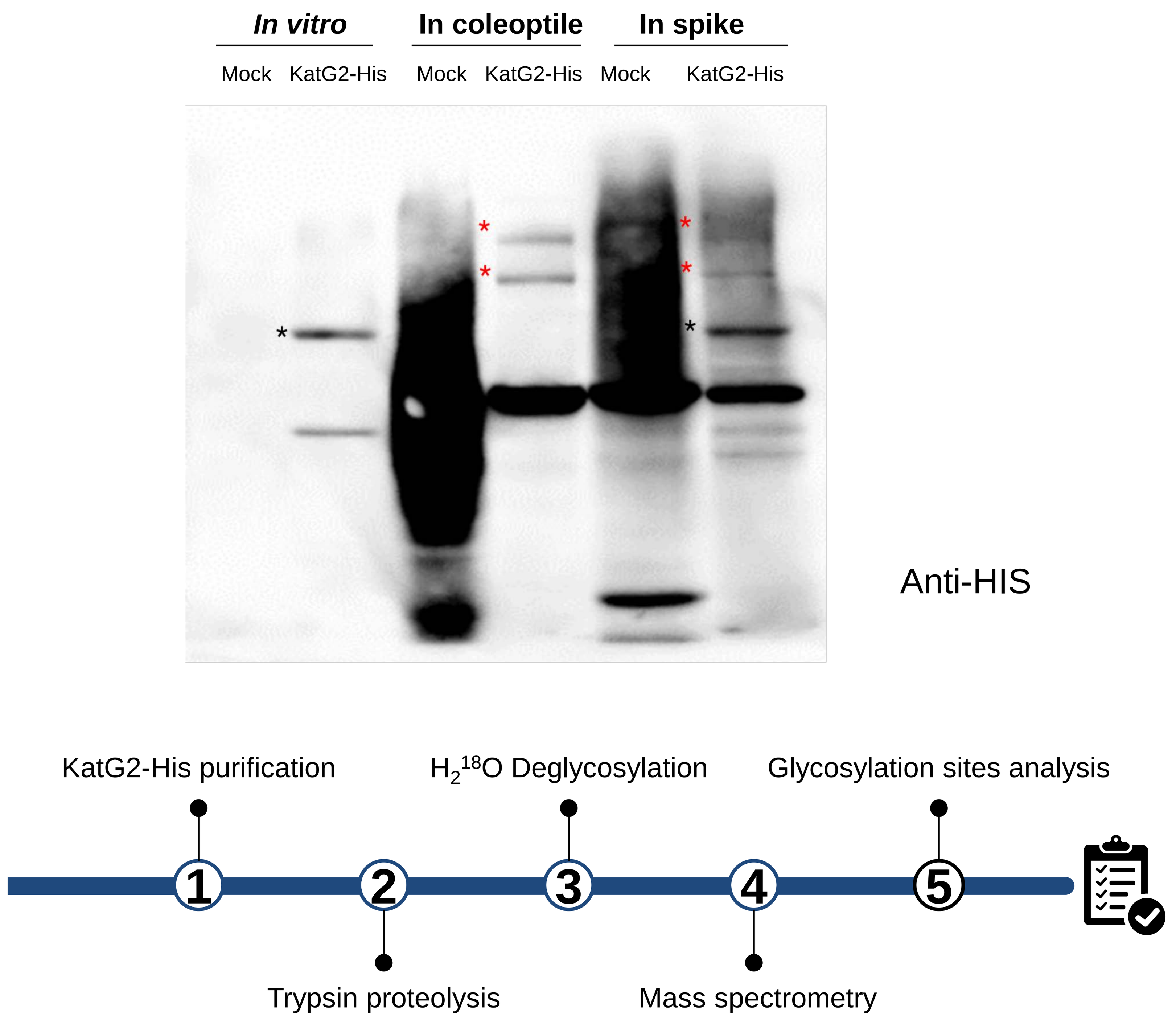

Fig. S7 N-glycosylation site identification of KatG2. (A) Western blot of purified protein from strain with constitutive promoter-driven KatG2-His-tagged fusion growing on a V8 agar plate *in vitr*o, in wheat coleoptile, and in wheat spike. The black asterisk indicates KatG2 without modification, and the red asterisk indicates the predicted KatG2 modified with glycosylation. (B) Schematic diagrams show the process of detecting the *N*-glycosylation with high-resolution LC-MS/MS.
