# Supplementary material for: The spatiotemporal control of KatG2 catalase‐peroxidase contributes to the invasiveness of Fusarium graminearum in host plants
Source: Mol Plant Pathol. 2019 Mar 27;20(5):685–700. doi: 10.1111/mpp.12785 (PMC6637876; doi:10.1111/mpp.12785)
Supplement: Supplementary file 8 — Fig. S8 N glycosylation sites of KatG2 localized at the surface of the KatG2 tertiary structure. Homology modelling of KatG2 using the SWISS MODEL web server. KatG2 forms a homodimer, and the yellow sites indicate the asparagines at positions 238 and 391, which were predicted to be modified with N glycosylation. [file MPP-20-685-s008.pdf]

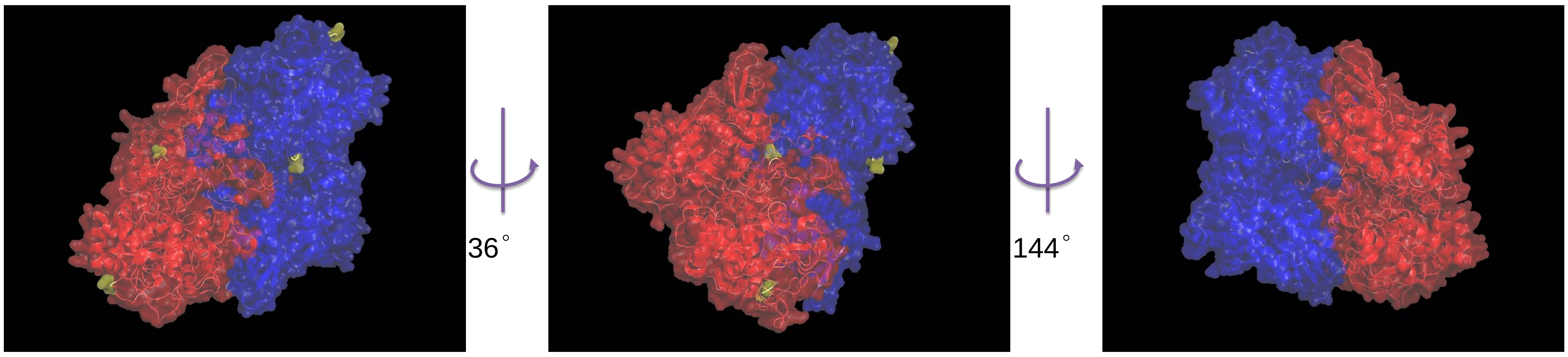

Fig. S8 N-glycosylation sites of KatG2 localized at the surface of the KatG2 tertiary structure. Homology modeling of KatG2 using the SWISS-MODEL web server. KatG2 forms a homodimer, and the yellow sites indicate the asparagines at positions 238 and 391, which were predicted to be modified with N-glycosylation.
