# Supplementary material for: The spatiotemporal control of KatG2 catalase‐peroxidase contributes to the invasiveness of Fusarium graminearum in host plants
Source: Mol Plant Pathol. 2019 Mar 27;20(5):685–700. doi: 10.1111/mpp.12785 (PMC6637876; doi:10.1111/mpp.12785)
Supplement: Supplementary file 9 — Fig. S9 Asparagines at positions 238 and 391 of KatG2 regulate the cell wall distribution of KatG2. (A) KatG2 mRFP localization in conidia with or without mutation of N glycosylation sites. To analyse the localization of KatG2 in conidia, the mutation sites were introduced to the plasmid in Fig. 4B, which was then transformed into PH 1 protoplasts. Bar = 30 μm. (B) Statistical analysis of KatG2 localization using AmCyan as a reference. Error bars indicate standard deviation, the experiment was repeated three times, and asterisks indicate significant differences (Student’s t‐test, P < 0.05). [file MPP-20-685-s009.pdf]

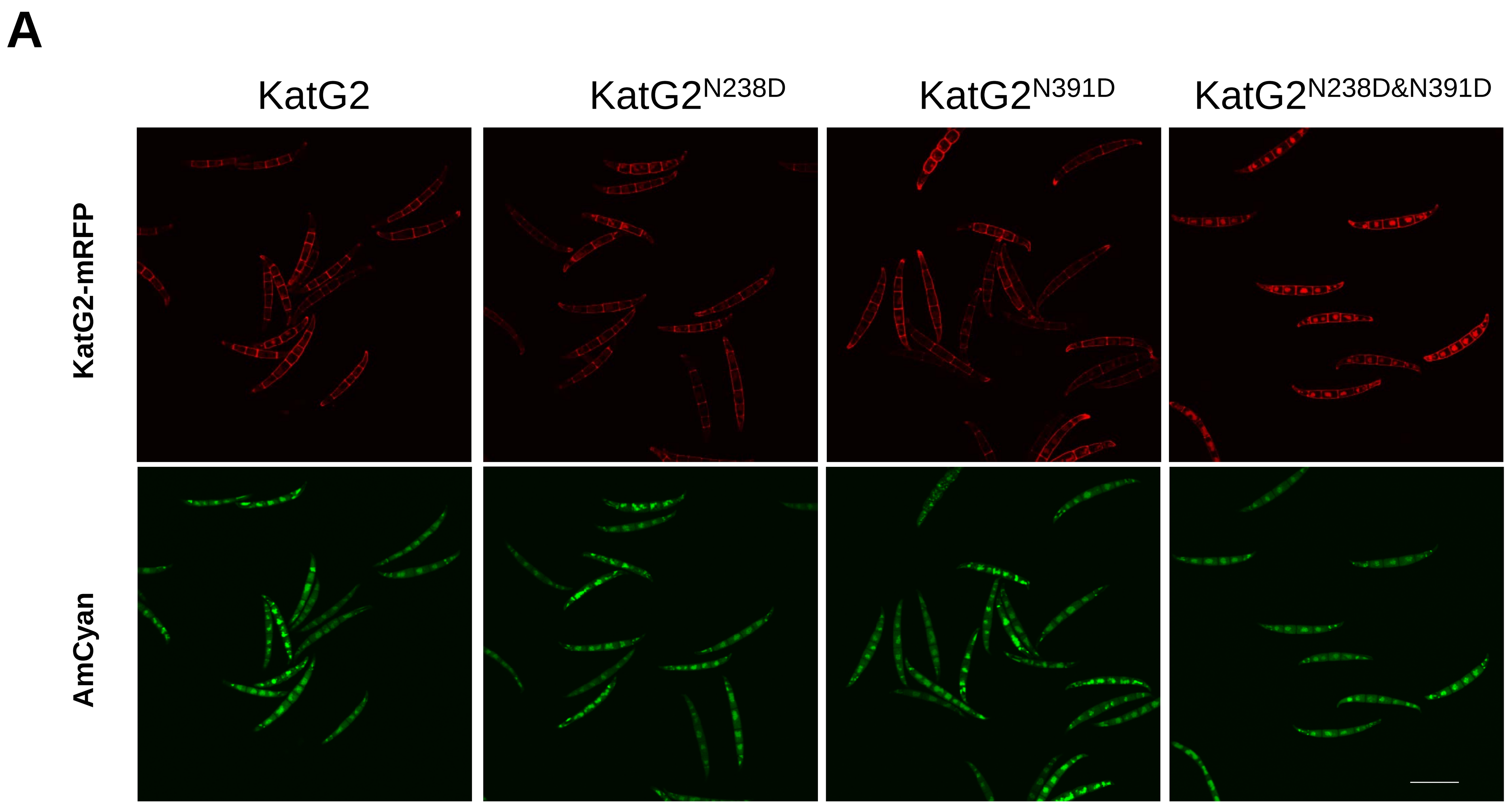

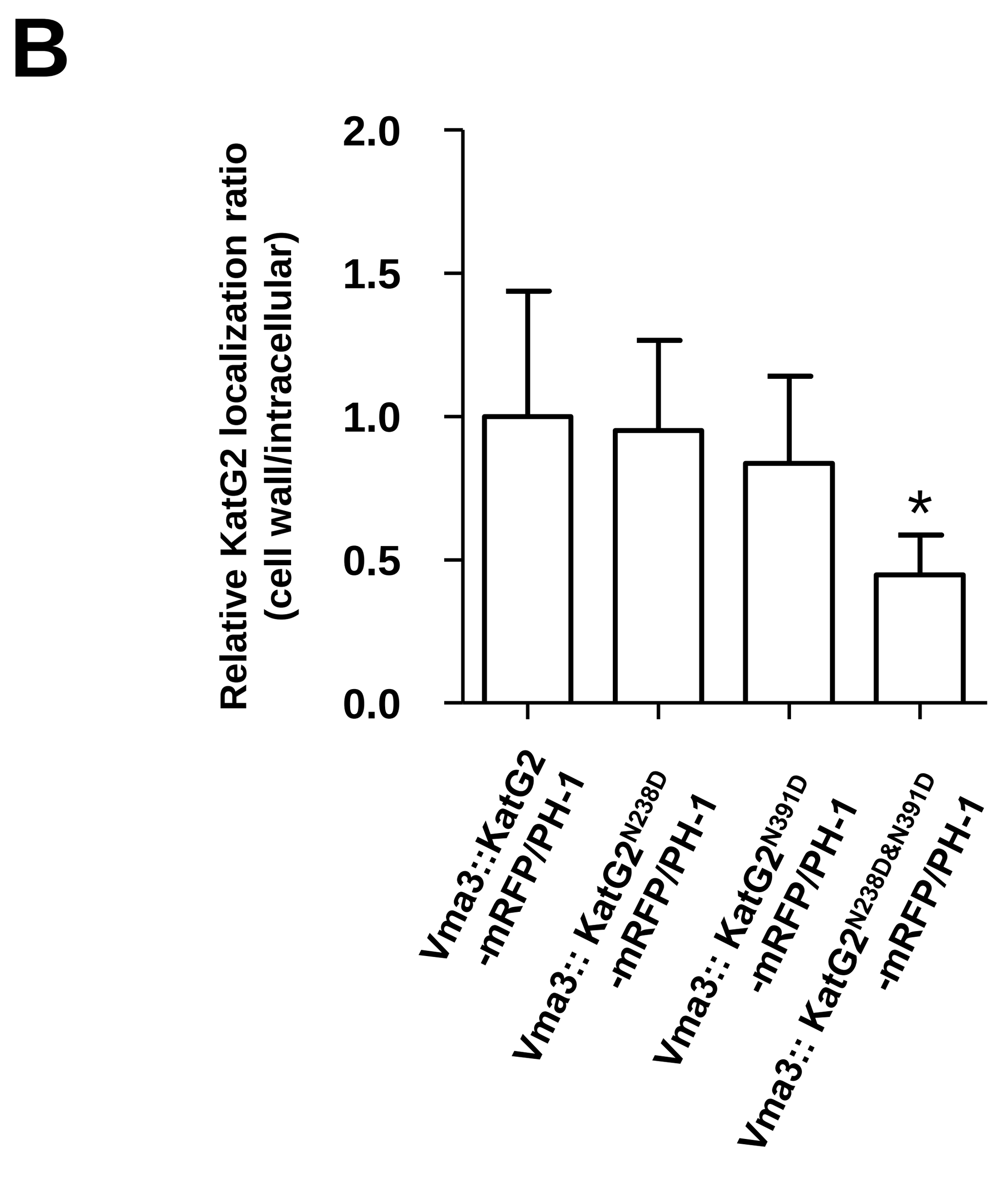

Fig. S9 Asparagine residues at positions 238 and 391 of KatG2 regulate the cell wall distribution of KatG2. (A) KatG2-mRFP localization in conidia with or without mutation of N-glycosylation sites. To analyze the localization of KatG2 in conidia, the mutation sites were introduced to the plasmid in Fig. 4B, which was then transformed into PH-1 protoplasts. Bar=30 μm. (B) Statistical analysis of KatG2 localization using AmCyan as a reference. Error bars indicate standard deviation, the experiment was repeated three times, and asterisks indicate significant differences (Student's *t*-test P<0.05).
