# Supplementary material for: The spatiotemporal control of KatG2 catalase‐peroxidase contributes to the invasiveness of Fusarium graminearum in host plants
Source: Mol Plant Pathol. 2019 Mar 27;20(5):685–700. doi: 10.1111/mpp.12785 (PMC6637876; doi:10.1111/mpp.12785)
Supplement: Supplementary file 10 [file MPP-20-685-s010.pdf]

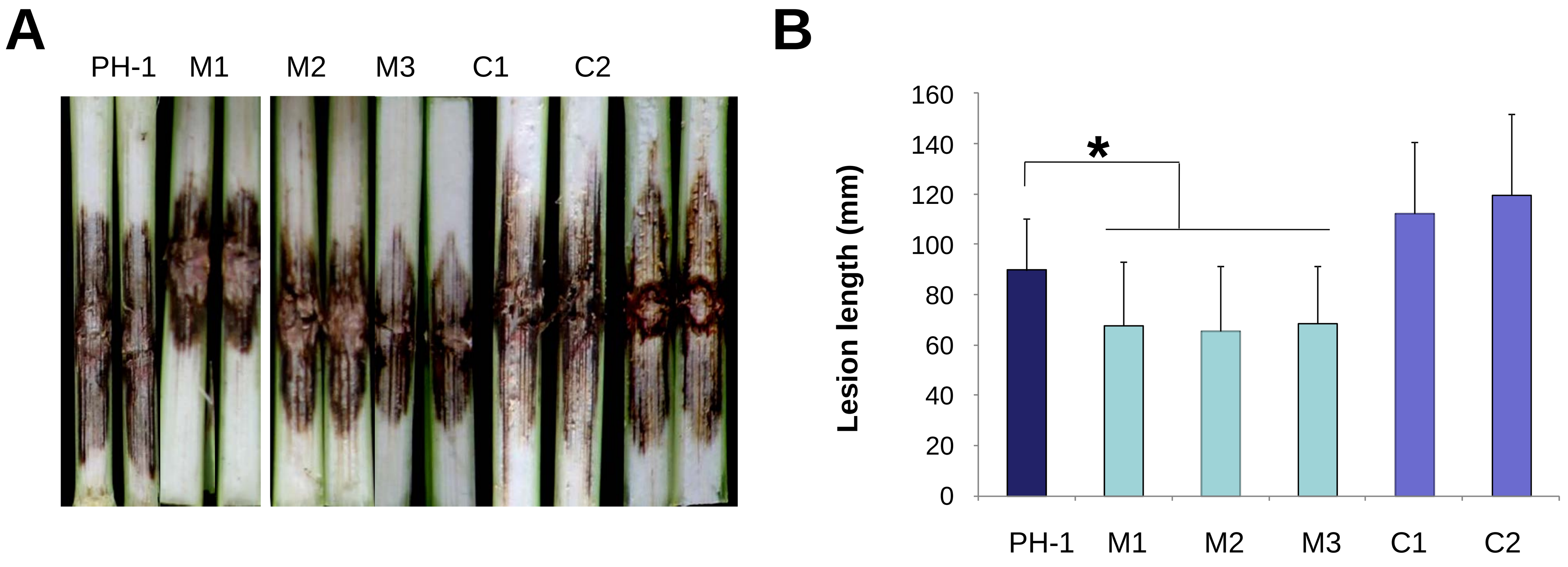

Fig. S10 Assays of Δ*KatG2* infection on maize stalks. (A) Diseased maize stalks inoculated with PH-1, Δ*KatG2* mutants and complemented strains at 14 dpi. (B) Lesion length of infected stalks at 14 dpi. Error bars indicate standard errors derived from three independent experiments. Student's *t*-test $P<0.05$.
