# Supplementary material for: The spatiotemporal control of KatG2 catalase‐peroxidase contributes to the invasiveness of Fusarium graminearum in host plants
Source: Mol Plant Pathol. 2019 Mar 27;20(5):685–700. doi: 10.1111/mpp.12785 (PMC6637876; doi:10.1111/mpp.12785)
Supplement: Supplementary file 11 [file MPP-20-685-s011.docx]

**Table S1. Five catalase and two catalase-peroxidase genes in *F. graminearum* genome**

| **Gene ID** | **Name** | **Type** | **Protein size** | **MW (kDa)/pI** | **Localization**  **prediction** | **Gene Name in reference (Lee et al., 2014)** |
| --- | --- | --- | --- | --- | --- | --- |
| FGSG_02881 | *CAT1* | Small subunit monofunctionalcatalase | 532 | 60.3/7.3 | Peroxisomal | *FCA4* |
| FGSG_06596 | *CAT2* | Small subunit monofunctionalcatalase | 584 | 66.3/5.9 | Peroxisomal | *FCA5* |
| FGSG_16526 | *CAT3* | Small subunit monofunctionalcatalase | 576 | 65.2/6.4 | Peroxisomal | *FCA3* |
| FGSG_06733 | *CAT4* | Large subunit monofunctionalcatalase | 716 | 78.6/6.1 | Secreted/extracellular space | *FCA2* |
| FGSG_06554 | *CAT5* | Large subunit monofunctionalcatalase | 738 | 82.7/5.8 | Cytoplasmic | *FCA1* |
| FGSG_02974 | *KatG1* | Bifunctional catalase | 801 | 88.5/6.5 | Mitochondrial | *FCA6* |
| FGSG_12369 | *KatG2* | Bifunctional catalase | 777 | 85.5/6.3 | Secreted/cell wall | *FCA7* |
