# Supplementary material for: The spatiotemporal control of KatG2 catalase‐peroxidase contributes to the invasiveness of Fusarium graminearum in host plants
Source: Mol Plant Pathol. 2019 Mar 27;20(5):685–700. doi: 10.1111/mpp.12785 (PMC6637876; doi:10.1111/mpp.12785)
Supplement: Supplementary file 12 [file MPP-20-685-s012.docx]

**Table S2. Primers used in this study**

| **Primers** | **Sequences (5’-3’)** | **Purpose** |
| --- | --- | --- |
| KOF1/R1 | ATTGGACCACCTACGGGCAC/  TAGCCACGATTCGAAGCCGCAGTTTTGTAGGCAGCGGGAG | *KatG2* gene knock out |
| KOF2/R2 | CGCATTGAATTGAAAAAGGAAGAGTATGAAGGTGAGCAAGTAGAGAC  GAAATGTAGAAACGGCGAGA | *KatG2* gene knock out |
| HYF/R | GCGGCTTCGAATCGTGGCTA/  GTATTGACCGATTCCTTGCGGTCCGAA | *HPH* gene amplification |
| YGF/R | GATGTAGGAGGGCGTGGATATGTCCT/  CATACTCTTCCTTTTTCAATTCAATTCAATGCG | *HPH* gene amplification |
| INF/INR | CCAAGCGTGATACCCAGCAG/  TTCAGTGGGGGTAGCGAGAC | △*KatG2* mutants verification |
| UF/DR | CTCCCGCTGCCTACAAAACT/  GTCTCTACTTGCTCACCTT | △*KatG2* mutants verification |
| ComF1/R1 | TCCACTAGTTCTAGAGCGGCCGCCGATTGGACCACCTACGGG/  AACGATCTGCAGCCGGGCAAGTGACTCCCTAACCA | *KatG2* gene complementation |
| FluF1/R1 | AAAACTAGTATGCACGCCAAAACTCTCTT/  CGCGGATCCACTTGCTCACCTTGACGTCGA | Vma3::KatG2-mRFP construct |
| 28aF/R | AGCAAATGGGTCGCGGATCCATGCACGCCAAAACTCTCTT  TGGTGCTCGAGTGCGGCCGCCTTGCTCACCTTGACGTCGAAG | *KatG2*  expression vector |
| ProbeAF/R | CCAAGCGTGATACCCAGCAG/  TTCAGTGGGGGTAGCGAGAC | Probe A vector |
| ProbeBF/R | GTCGGTTTCCACTATCGGCGAGTA/  ACTGGCAAACTGTGATGGACGACA | Probe B vector |
| RTF/R | GCCAAGCGAGGATTCCACAT/  CGTCAGTCTCAACGTTTCCG | Real-time PCR of *KatG2* |
| TubF/R | AGGTTGAGGACCAGATGCG/  CCTGGATAGAGGTGGAGTTTC | Real-time PCR reference gene |
| KIF/KIR | CTCCACCGTGATCTCGGGAC/  AGAAACGGCGAGAGGTGTGC | PKatG2:: KatG-mRFP verification |
