# Supplementary material for: The spatiotemporal control of KatG2 catalase‐peroxidase contributes to the invasiveness of Fusarium graminearum in host plants
Source: Mol Plant Pathol. 2019 Mar 27;20(5):685–700. doi: 10.1111/mpp.12785 (PMC6637876; doi:10.1111/mpp.12785)
Supplement: Supplementary file 13 [file MPP-20-685-s013.docx]

**Table S3. Accession number of CATs and KatGs used Figure S1**

| **Fungus** | **Gene** | **NCBI accession number** |
| --- | --- | --- |
| *Aspergillus fumigatus* | AfCAT3 | XP_749156.1 |
|  | AfCAT2 | XP_756140.1 |
| *Aspergillus nidulans* | AnCATC | XP_663522 |
|  | AnCATD | XP_681822 |
| *Bipolaris maydis* | BmCAT1 | AAR17472.1 |
| *Saccharomyces cerevisiae* | ScCTT1 | NP_011602 |
|  | ScCATA | NP_010542 |
| *Neurospora crassa* | NcCAT2 | XP_961873 |
| *Cryptococcus neoformans* | CnCAT2 | ABG26353.1 |
| *Candida albicans* | CaCAT1 | XP_718734 |
| *Cryptococcus neoformans* | CnCAT1 | ABG26352 |
|  | CnCAT3 | ABG26354.1 |
|  | CnCAT4 | ABG26355 |
| *Bipolaris maydis* | BmCAT2 | AAR17473.1 |
|  | BmCAT3 | AAR17474.1 |
| *Aspergillus fumigatus* | AfCAT1 | XP_748550.1 |
|  | AfCATA | XP_747688 |
| *Aspergillus nidulans* | AnCATA | XP_681906 |
|  | AnCATB | XP_692608 |
| *Magnaporthe oryzae* | MoCATA | XP_003717445 |
|  | MoCATB | XP_003717126 |
| *Neurospora crassa* | NcCAT1 | XP_956234 |
|  | NcCAT3 | XP_957826 |
| *Fusarium oxysporum* | FoCP01 | KNB12728.1 |
|  | FoCP02 | KNB19974.1 |
|  | FoCP03 | KNB15903 |
|  | FoCP04 | KNB20204.1 |
|  | FoCP05 | KNB19949.1 |
| *Colletotrichum graminicola* | CgKatG1 | XP_008094697.1 |
|  | CgKatG2 | XP_008098502.1 |
| *Magnaporthe oryzae* | MoKatG1 | XP_003719470 |
|  | MoKatG2 | XP_003720407 |
| *Ustilago maydis* | UmKatG | XP_011390075 |
| *Aspergillus nidulans* | AnKatG | XP_680657 |
| *Aspergillus fumigatus* | AfKatG | XP_747039 |
| *Neurospora crassa* | NcKatG | XP_959745.1 |
