# Supplementary material for: The spatiotemporal control of KatG2 catalase‐peroxidase contributes to the invasiveness of Fusarium graminearum in host plants
Source: Mol Plant Pathol. 2019 Mar 27;20(5):685–700. doi: 10.1111/mpp.12785 (PMC6637876; doi:10.1111/mpp.12785)
Supplement: Supplementary file 14 [file MPP-20-685-s014.docx]

**Table S4. *F. graminearum* strains used in this study**

| **Strain** | **Characteristics** | **Source/Reference** |
| --- | --- | --- |
| *PH-1* | *Fusarium graminearum* PH-1 (NRRL 31084) | Lab stock |
| *AmCyanPH-1* | *vma3prom:AmCyan*/*PH-1* | Zhang et al., 2012 |
| *ΔKatG2* | KatG2 deletion of *PH-1* | This work Fig. S2 |
| *AmCyan∆KatG2* | *vma3prom:AmCyan/∆KatG2* | This work |
| *pKatG2:KatG2-mRFP* | *KatG2prom:KatG2-mRFP/PH-1*  (native *KatG2* promoter, mRFP knock-in) | This work |
| *KatG2-mRFP* | *vma3prom:KatG2-mRFP/PH-1* | This work Fig. 5b |
| *pvma3:KatG2-mRFP; AmCyan* | *vma3prom:KatG2-mRFP;EF-alphaprom:AmCyan/PH-1* (constitutive promoter driven *KatG2*) | This work |
| *pvma3:KatG2^N238D^-mRFP/PH-1* | *vma3prom: KatG2^N238D^-mRFP EF-alphaprom:AmCyan /PH-1* | This work |
| *pvma3: KatG2^N391D^-mRFP/PH-1* | *vma3prom:KatG2^N391D^-mRFP; EF-alphaprom:AmCyan /PH-1* | This work |
| *KatG2^N238D&N391D^-mRFP/PH-1* | *vma3prom: KatG2^N238D&N391D^-mRFP; EF-alphaprom:AmCyan /PH-1* | This work |
| *KatG2^N238D&N391D^-mRFP/∆KatG2* | *vma3prom: KatG2^N238D&N391D^-mRFP/∆KatG2* | This work |
| *pvma3:KatG2^N238D&N391D^-mRFP; AmCyan/ΔKatG2* | *vma3pro:KatG2^N238D&N391D^-mRFP; EF-alphaprom:AmCyan/ΔKatG2*  (constitutive promoter driven double mutant KatG2-mRFP) | This work |
| *KatG2-His* | *vma3prom:KatG2-His_6_/PH-1* | This work |
